# Kinsenoside‐Loaded Microneedle Accelerates Diabetic Wound Healing by Reprogramming Macrophage Metabolism via Inhibiting IRE1α/XBP1 Signaling Axis

**DOI:** 10.1002/advs.202502293

**Published:** 2025-04-25

**Authors:** Li Lu, Jiewen Liao, Chao Xu, Yuan Xiong, Juan Zhou, Guangji Wang, Ze Lin, Kangkang Zha, Chuanlu Lin, Ruiyin Zeng, Guandong Dai, Qian Feng, Bobin Mi, Guohui Liu

**Affiliations:** ^1^ Department of Orthopedics Union Hospital, Tongji Medical College Huazhong University of Science and Technology Wuhan 430022 China; ^2^ Department of Rehabilitation Tongji Hospital, Tongji Medical College Huazhong University of Science and Technology Wuhan 430030 China; ^3^ Hubei Province Key Laboratory of Oral and Maxillofacial Development and Regeneration Wuhan 430022 China; ^4^ Key Laboratory of Biorheological Science and Technology Ministry of Education College of Bioengineering Chongqing University Chongqing 400044 China; ^5^ Department of Orthopedics Tongji Hospital, Tongji Medical College Huazhong University of Science and Technology Wuhan 430030 China; ^6^ Department of Cardiology Hubei Provincial Hospital of Traditional Chinese Medicine Wuhan 430073 China; ^7^ Department of Cardiology The Central Hospital of Wuhan, Tongji Medical College Huazhong University of Science and Technology Wuhan 430073 China; ^8^ Department of Orthopaedics Pingshan District People's Hospital of Shenzhen Pingshan General Hospital of Southern Medical University Shenzhen Guangdong 518118 China

**Keywords:** diabetic wound, inflammation, kinsenoside, macrophage, metabolic reprogramming, microneedle

## Abstract

Continuously bacterial infection, undue oxidative stress, and inflammatory responses in the skin tissue microenvironment determine the delayed healing outcome of diabetic wounds, which remain a tough clinical challenge and need multifaceted therapeutic strategies. In this work, HA‐ADH/HA‐QA‐ALD‐based hydrogel microneedle (HAQA‐MN) with antimicrobial and antioxidative activities incorporating kinsenoside (KD) coated with macrophage membrane (M‐KD) targeting inflammation relief is developed to improve the cutaneous micro‐niche. KD is observed to trigger trimethylamine N‐oxide‐irritated proinflammatory macrophages repolarization from M1 state to anti‐inflammatory M2 phenotype, and the underlying mechanism is due to drug‐induced IRE1α/XBP1/HIF‐1α pathway suppression, accompanied by diminution of glycolysis and enhancement of oxidative phosphorylation, resulting in proinflammatory cascade inhibition and anti‐inflammatory signaling enhancement. The hydrazone cross‐linked HAQA‐MN possesses favorable biocompatibility, self‐healing, controlled release of M‐KD and excellent mechanical properties. Moreover, the MN patch remarkedly restrains the survival of *E. coli* and *S. aureus* and eliminates hydrogen peroxide to preserve cellular viability. Notably, M‐KD@HAQA‐MN array effectively ameliorates cutaneous inflammation and oxidative stress and facilitate angiogenesis and collagen deposition, thereby accelerating tissue regeneration of diabetic mice with a full‐thickness skin defect model. Collectively, this study highlights a multifunctional MN platform as a promising candidate in clinical application for the treatment of diabetic wounds.

## Graphical abstract

Gut metabolite trimethylamine N‐oxide accumulates in the diabetic wound area to amplify macrophage inflammation via enhancing glycolysis activities. Kinsenoside induces macrophage repolarization from M1 to M2 phenotype through inhibiting IRE1α/XBP1 pathway, followed by HIF‐1α‐glycolysis axis repression and mitophagy‐oxidative phosphorylation axis activation. Hyaluronic acid‐based hydrogel microneedle encapsulating macrophage membrane‐coated kinsenoside accelerates diabetic wound healing via multifunctional merits and enhanced drug bioavailability.

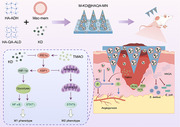

## 1 Introduction

Given that the unhealthy lifestyles including high calorie diet, lack of exercise and excessive tiredness affect more and more individuals currently, the incidence of diabetes is on the rise year by year worldwide.^[^ ^1^ ^]^ It has been demonstrated that diabetic foot ulcer (DFU), with a prevalence reaching ≈20 million per year, is one of the most common complications of diabetes and is responsible for enormous economy and health‐care burden.^[^ ^2^, ^3^ ^]^ Treatment approaches for DFU like surgical debridement, antibacterial dressing, negative pressure application and stem cell transplantation have been utilized, whereas the therapeutic effects are still not satisfactory.^[^ ^4^, ^5^ ^]^ Therefore, seeking alterative and cost‐effective approaches with highly efficiency and safety antagonizing DFU are urgent.

Macrophages are particularly indispensable in cutaneous wound regeneration because of their bilateral regulatory roles.^[^ ^6^ ^]^ Considering that M2 macrophages are capable of releasing anti‐inflammatory and pro‐regenerative cytokines such as transforming growth factor‐β (TGF‐β), interleukin 4 (IL‐4) and vascularendothelial growth factor A (VEGFA) to promote angiogenesis and collagen disposition, phenotype switching obstacle from M1 to M2 of macrophages induced by persistent inflammation stimulation enables the diabetic wound healing process to stagnate at the prolonged inflammatory phase, leading to the M1 macrophages continuously secrete inflammation agents including tumor necrosis factor α (TNF‐α), monocyte chemotactic protein‐1 (MCP‐1) and IL‐6 to impede new vessel formation and slow down tissue repair.^[^ ^7^ ^]^ With respect to the molecular mechanisms underlying phenotype regulation, metabolic pattern conversion is deemed to be implicated.^[^ ^8^ ^]^ Cumulative evidences have established that glycolysis dominates the energy production process in M1 population, whereas oxidative phosphorylation (OXPHOS) is the leading metabolic mode in M2 cohort.^[^ ^9^ ^]^ Although the M1 directional tilting has been documented in the diabetic wound tissue, the dominant metabolic path in macrophages is poorly understood.^[^ ^10^ ^]^

Trimethylamine N‐oxide (TMAO) is known as a kind of metabolic product of gut microbiota. Choline, betaine and L‐carnitine‐containing dietary ingredients are metabolized by intestinal flora to generate trimethylamine, the latter then enters the peripheral circulation and undergoes oxidation to form TMAO in the liver.^[^ ^11^, ^12^ ^]^ Serum TMAO concentration is proved to be closely associated with the development of inflammation‐primed diseases like atherosclerosis, heart failure and neurodegeneration.^[^ ^13^, ^14^, ^15^ ^]^ More intriguingly, recent clinical studies have revealed the high level of circulating TMAO in diabetes individuals.^[^ ^16^ ^]^ Considering that mounting evidence strongly supports the intimately involvement of TMAO in inflammation progression of macrophages, there is a possibility that TMAO accumulates in the diabetic wound area to facilitate the dysfunction of immune microenvironment via enhancing the polarization of pro‐inflammatory M1 stage.^[^ ^17^, ^18^ ^]^ Elucidating the intrinsic interaction between TMAO and macrophage phenotype conversion is worthy for further recognizing potential targets required for the inflammation extension in DFU tissues.

Isolated from the *Anoectochilus roxburghii*, kinsenoside (KD) is found as a bioactive compound possessing potent anti‐inflammatory properties.^[^ ^19^ ^]^ It was revealed that KD restrained the phosphatidylinositol‐3‐kinase (PI3K)/protein kinase B (AKT) signal pathway to repress the maturation of dendritic cells and subsequent inflammation progression in the liver tissue.^[^ ^20^ ^]^ Additionally, KD was uncovered to disrupt NOD‐like receptor protein 3 (NLRP3) inflammasome assembly and IL‐1β generation of macrophages via abrogating nuclear factor κB (NF‐κB) cascade.^[^ ^21^ ^]^ Via enhancing the inhibitor κB (IκB) activity and lowering c‐Jun N‐terminal kinase (JNK) and p38 activation, KD facilitated the polarized signature skewing toward M2 state of macrophages, which was beneficial for damage relief of chondrocytes.^[^ ^22^ ^]^ Owing to the pronounced anti‐inflammatory ability, KD is likely to trigger macrophages polarization switching from M1 to M2 phenotype to promote diabetic wound healing. However, the quick release and lower retention of phytochemicals in local area restrict their application, increasing the bioavailability of KD is of high priority.

**Scheme 1:**
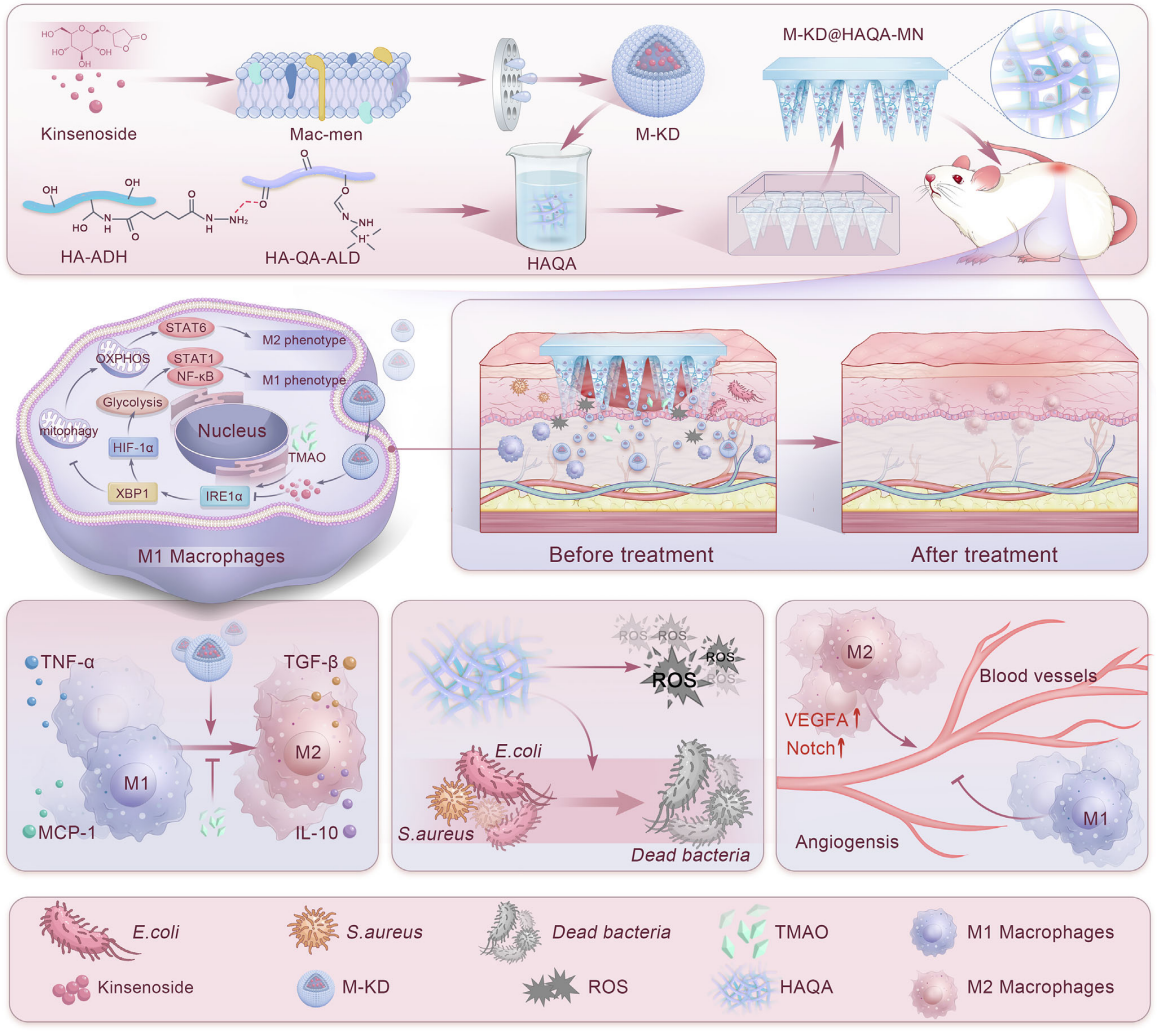
Schematic illustration of the preparation of M‐KD@HAQA‐MN and its beneficial roles in facilitating diabetic wound healing.

In this study, HA‐ADH/HA‐QA‐ALD‐based hydrogel (HAQA) possessing excellent biocompatibility, self‐healing, 3D porous network and controlled release capacities was introduced as a drug vehicle. Due to the high plasticity, the appearance of the HAQA hydrogel was shaped into a microneedle (MN) structure with robust mechanical strain capable of penetrating the epidermis facilely, promoting entrance of KD into the deep tissue. Meanwhile, the surface of KD was coated with macrophage membrane layer (M‐KD) conferred target cell affinity on the compound, enhancing KD‐induced macrophage inflammation amelioration via reprogramming glucose metabolism. Importantly, the novel M‐KD@HAQA‐MN system effectively suppressed inflammation, inhibited bacterial growth, neutralized oxidative stress, facilitated angiogenesis and then promoted wound healing in a diabetic mouse model. This study highlighted the potential of the M‐KD‐loaded HAQA‐MN platform as a multifaceted therapeutic approach for managing diabetic wounds, offering a promising avenue for future clinical applications (**Scheme** **1**).

## 2 Results

### 2.1 The Conjunction of TMAO Abundance and Metabolism Alteration in the Inflammatory Microenvironment of Diabetic Wounds

**Figure 1:**
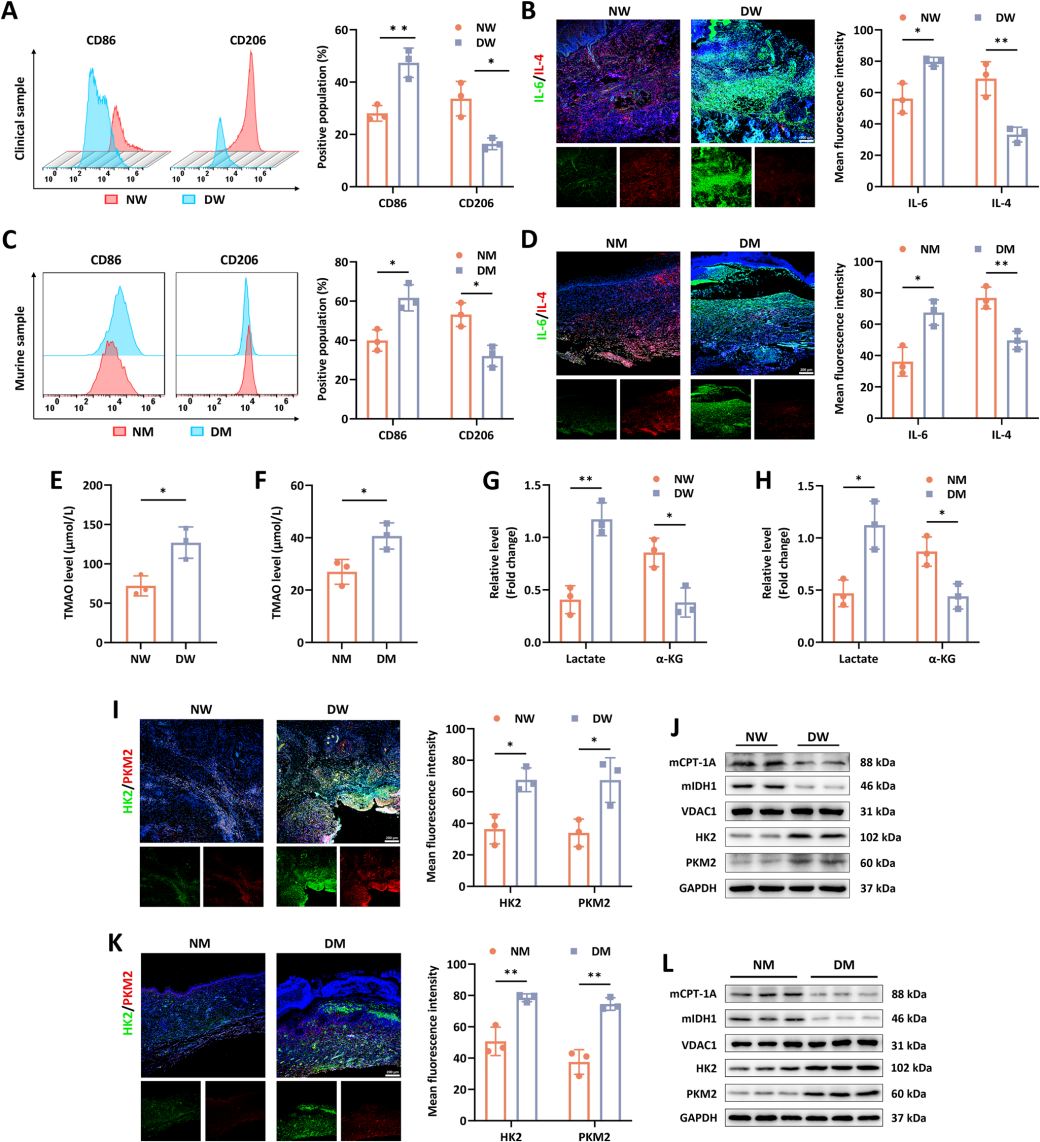
The inflammatory and metabolic profiles in diabetic and normal wound tissues. A) The percentage of CD86+ and CD206+ cells among the CD11b+F4/80+ macrophages in the normal wound (NW) and diabetic wound (DW) of patients (*n* = 3). B) The expression of IL‐6 and IL‐4 in the NW and DW tissue of patients (*n* = 3). C) Flow cytometry of CD86 and CD206 positive population in the CD11b+F4/80+ cells from the wound tissue of the normal mice (NM) and diabetic mice (DM) at day 7 post‐operation (*n* = 3). D) Immunofluorescence staining of IL‐6 and IL‐4 in the wound area of the NM and DM group (*n* = 3). E) The concentration of TMAO in the normal and diabetic wound tissues from patients and F) mice (*n* = 3). G) The relative level of lactate and α‐KG in the skin wound tissues from patients and H) mice (*n* = 3). I) The level of HK2 and PKM2 in the wound area of the patient groups (*n* = 3). J) Protein contents of HK2, PKM2, mitochondrial IDH1 and CPT‐1A in the NW and DW tissues (*n* = 4). K) Immunofluorescence staining showed the level of HK2 and PKM2 in the wound area from the NM and DM (*n* = 3). L) Western blot analysis was performed to determine the expression of HK2, PKM2, mitochondrial IDH1 and mitochondrial CPT‐1A in the NM and DM wound tissues (*n* = 3). Data were presented as mean ± SD. Statistical significance was determined using Student's *t*‐test. *p < 0.05, **p < 0.01.

It has been documented that equilibrium failure of macrophage polarization induced by compromised immunomodulation is considered as a pathological feature of the diabetic wound niche, leading to improper accumulation of M1 and scarcity of M2 macrophages.^[^ ^6^ ^]^ As expected, we observed that the percentage of CD86+ M1 macrophages was significantly increase in the wound tissue of DFU patients than that in the wound site from foot trauma patients without underlying disease, yet the ratio of CD206+ M2 macrophages was decreased in the diabetic wound compared to the normal wound (**Figure** **1**A). In light of the dominant roles of overactive macrophages‐induced immune homeostasis imbalance in halting the transition of different stages among wound healing,^[^ ^23^ ^]^ we further investigated the inflammation factors and discovered that there was a level elevation of IL‐6 responsible for immunocytes activation in the diabetic cutaneous tissue and an increase in the expression of IL‐4 involved in inflammation resolution was seen in normal wound area (Figure 1B). Meanwhile, we observed that wound tissues of the diabetic mice displayed high M1 to M2 ratio compared to the normal wound, as manifested by increased level of CD86 and reduced content of CD206 (Figure 1C). Similarly, cutaneous wound in the control group encountered the expression decrease of IL‐6 and level elevation of IL‐4 (Figure 1D). Growing evidence illustrates the contributing roles of TMAO in the progression of microvascular complications of diabetes, whereas the involvement of TMAO in the repair process of DFU is still elusive.^[^ ^17^, ^24^ ^]^ Herein, we found that the level of TMAO in the diabetic wound tissue was significantly elevated than the normal group (Figure 1E,F). Given that TMAO accelerated inflammation development of multiple tissues in vivo, we speculated that high content of TMAO participated in the delayed healing process of diabetic wounds depending on inflammation maintenance in local area.

Laboratory statistics have demonstrated that the pro‐inflammatory pathological microenvironment impels glycolysis which surpasses oxidative phosphorylation to dominate the energy metabolism, an underlying mechanism implicated in macrophage polarization abnormality.^[^ ^25^ ^]^ In this study, our findings showed that the wound tissue from diabetic samples experienced high level of glycolytic metabolite lactate, whereas α‐ketoglutarate (α‐KG), a metabolic intermediate among OXPHOS, was detected to present elevated level in normal wounds (Figure 1G,H). Moreover, we found that glycolysis‐related rate‐limiting enzyme hexokinase 2 (HK2) and pyruvate kinase isozyme type M2 (PKM2) developed an increased expression and mitochondrial carnitine palmitoyltransferase 1A (CPT‐1A) and isocitrate dehydrogenase 1 (IDH1) required for tricarboxylic acid (TCA) cycle had a reduced content in the wound region of diabetic individuals than the normal cohort, corroborating with the results of wound skin tissues of mice, as seen by the immunofluorescence staining and western bolt analysis (Figure 1I–L). These findings implied that in the diabetic wound region, enhanced glycolytic metabolic pattern might facilitate the M2 conversion blockade and longstanding M1‐related inflammation responses, which disturbed angiogenesis and exacerbated hypoxia, thereby forming a vicious cycle detrimental to wound healing.

### 2.2 KD Reversed Inflammation and Glycolysis in Macrophages Induced by TMAO

Subsequently, due to the abundance of TMAO in the diabetic wound microenvironment, the effects of TMAO on regulating macrophage inflammation were investigated. Our results indicated that TMAO incubation for 24 h increased the proportion of CD86+ cell population and decreased the percentage of CD206+ cluster among bone marrow‐derived macrophages (BMDMs) (Figure S1A, Supporting Information). The immunofluorescence staining suggested that inducible nitric oxide synthase (iNOS) and IL‐6 were highly expressed and arginase 1 (Arg‐1) and IL‐4 were in a low‐level in BMDMs upon TMAO stimulation (Figure S1B,C, Supporting Information). Additionally, content elevation of MCP‐1 and TNF‐α and expression reduction of TGF‐β and IL‐10 were discerned in TMAO‐insulted BMDMs, further affirming TMAO‐evoked impulse on pro‐inflammatory M1 switching and prejudice against anti‐inflammatory M2 conversion (Figure S1D, Supporting Information). Directly or indirectly activation on inflammation‐related signal pathways has been reported to be the molecular mechanism by which TMAO initiates and accelerates inflammation responses in macrophages.^[^ ^12^, ^26^ ^]^ Western blot analysis uncovered that TMAO enhanced the activities of signal transducer and activator of transcription 1 (STAT1) and NF‐κB, whereas weakened the activity of STAT6 axis responsible for inflammation diminishment (Figure S1E, Supporting Information).

Considerable documentations disclose that highly active glycolysis is indispensable for the persistence of M1 phenotype, so the metabolic pattern change in TMAO‐induced macrophages was determined.^[^ ^27^ ^]^ We found that TMAO administration significantly enhanced the glucose consumption and lactate generation as well as restrained the production of α‐KG (Figure S2A, Supporting Information). An increase in extracellular acidification rate (ECAR) and a decrease in oxygen consumption (OCR) further verified the elevated aerobic glycolytic function and reduced OXPHOS activity in BMDMs stimulated with TMAO (Figure S2B,C, Supporting Information). Then, the expression of rate‐limiting enzymes involved in glucose metabolism was detected. Our findings showed that high levels of HK2, PKM2 and lactate dehydrogenase (LDHA) and depressed expressions of mitochondrial IDH1 were presented in macrophages insulted by TMAO, indicating that pathway activity of glycolysis exceeded that of OXPHOS (Figure S3A,B, Supporting Information). In view of the acceerative effects of inflammation cytokines like TNF‐α on driving glycolysis,^[^ ^28^ ^]^ TMAO acted as a trigger point to induce the positive feedback loop formation between glycolysis and M1 state polarization, implying the importance of loop interruption on antagonizing macrophage inflammation and reshaping the pro‐proliferative immune condition.

**Figure 2:**
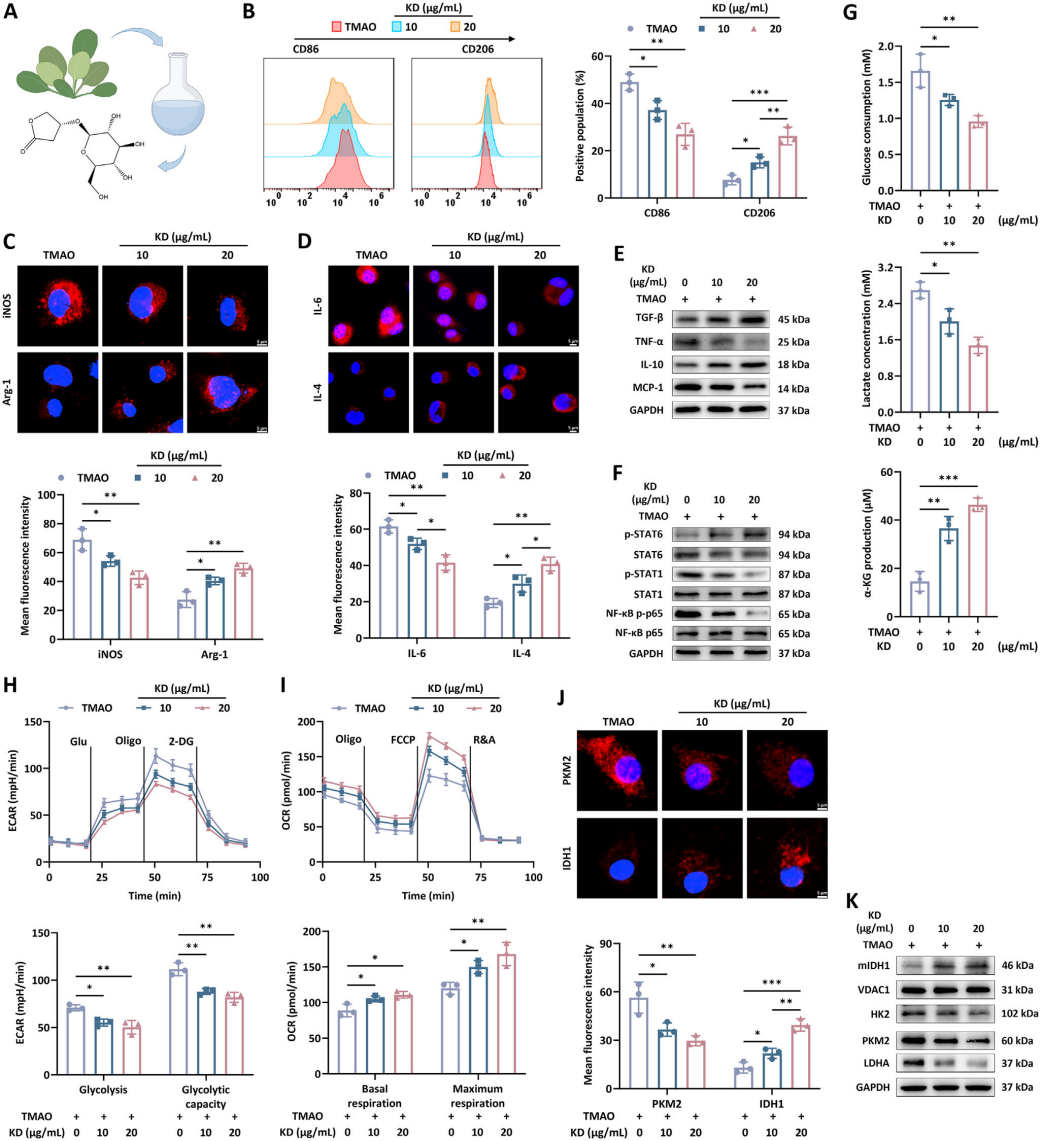
KD administration induced metabolic reprogramming and inflammation recession in macrophages. A) The chemical formula of KD. B) The percentage of CD86+ and CD206+ population in BMDMs (*n* = 3). C,D) Immunofluorescence staining and E) western blot was performed to analyze pro‐inflammatory and anti‐inflammatory factors in BMDMs with different treatments (*n* = 3). F) The activities of signaling pathway NF‐κB, STAT6 and STAT1 in BMDMs (*n* = 3). G) Following the indicated interventions, BMDMs were subjected to the detection of glucose consumption and lactate and α‐KG production (*n* = 3). H,I) The value of ECAR and OCR was determined using Seahorse assay (*n* = 3). J,K) The expression of glycolysis and TCA cycle‐related enzymes (*n* =3). Data were presented as mean ± SD. Statistical analysis was performed using one‐way ANOVA followed by Tukey's post hoc test. *p < 0.05, **p < 0.01, ***p < 0.001.

Belonging to the glycoside class, KD displays efficacious actions for managing metabolic syndrome, obesity and diabetes, with a pathophysiological character of rampant inflammation (**Figure** **2**A).^[^ ^19^ ^]^ To this end, involvement of KD in the regulation of macrophage inflammation induced by TMAO was assessed. Flow cytometry detection demonstrated a lower proportion of CD86+ M1 phenotype and a higher ratio of CD206+ M2 phenotype in TMAO‐provoked BMDMs following the treatment with KD (Figure 2B). Results from immunofluorescence assay manifested that TMAO‐elicited generation of iNOS and IL‐6 was dramatically lessened by KD. Instead, the expression of Arg‐1 and IL‐4 exhibited an increasing tread as the concentration of KD raised (Figure 2C,D). Consistently, measurement of protein levels via western blot unveiled depressed contents of pro‐inflammatory MCP‐1 and TNF‐α for the KD administration as well as enhanced expression of anti‐inflammatory TGF‐β and IL‐10 (Figure 2E). Cohesively, as delineated in Figure 2F, coincubation of KD notably counteracted TMAO‐primed activation of STAT1 and NF‐κB, while the signal transduction of STAT6 cascade was strengthened. Up to date, the regulatory roles of KD in glucose metabolism under pathological stimuli remains elusive. Herein, KD‐triggered glycolysis suppression and TCA cycle enhancement had been confirmed, as seen by the decline of glucose consumption, lactate generation and ECAR level as well as elevation of α‐KG production and high OCR, respectively in BMDMs (Figure 2G–I). Simultaneously, compared to low dosage of KD, this agent with high dosage yielded superior inhibitory effects on HK2, PKM2 and LDHA expression and robust increase of mitochondrial IDH1 level (Figure 2J,K). Therefore, these data suggested the prospect of KD for hampering the pro‐inflammatory metabolic pattern in macrophages and prompted us to explore the underlying mechanisms.

### 2.3 KD Regulated Metabolic Remodeling via Inhibiting IRE1α/XBP‐1 Pathway

**Figure 3:**
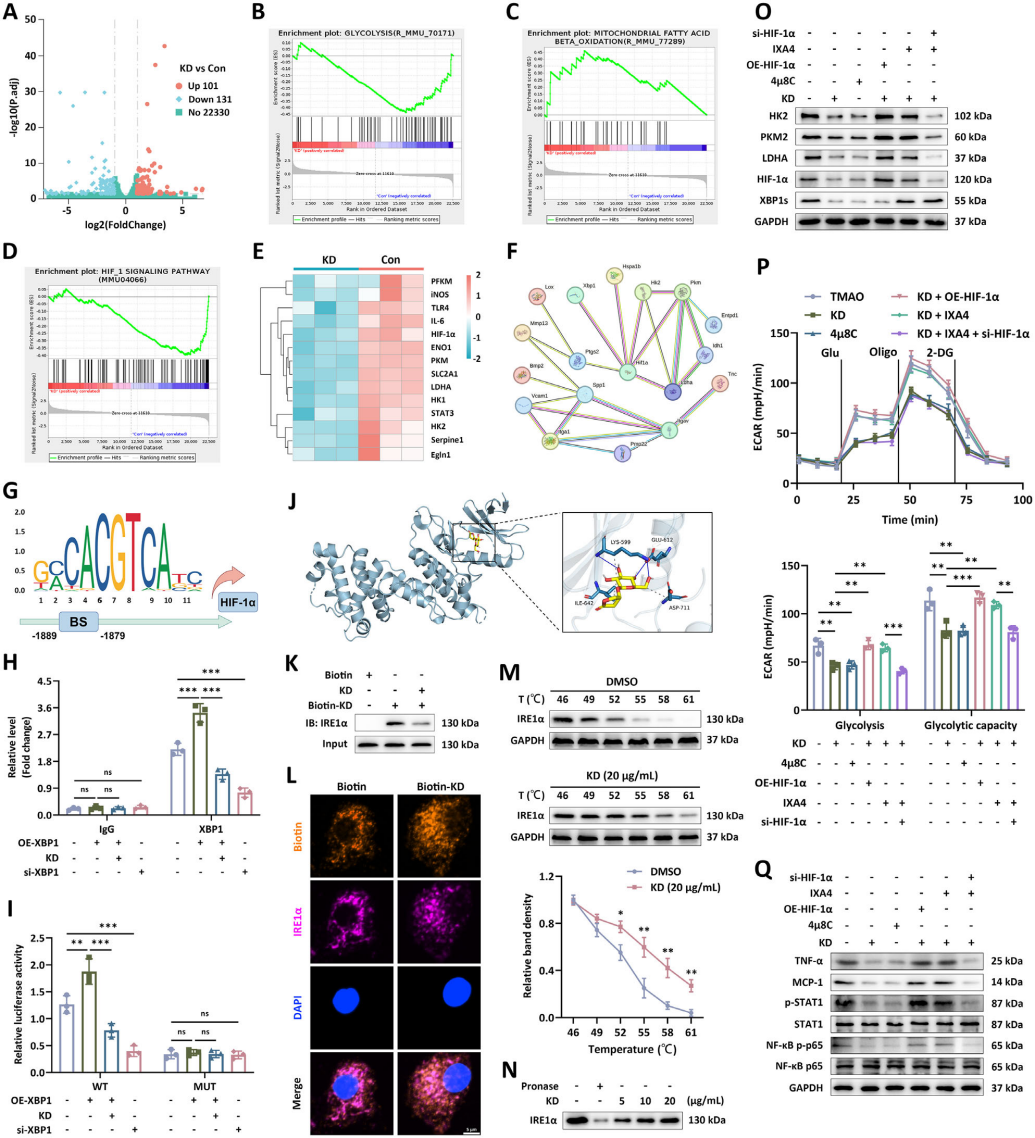
The mechanisms by which KD restrained glycolytic activities and downstream inflammatory responses in macrophages. A) The volcano plot between TMAO‐insulted BMDMs (Con) and TMAO‐insulted BMDMs with KD treatment (KD), *p* < 0.05, log2(fold change) >2. B,C) GSEA enrichment of glycolysis and mitochondrial fatty acid β oxidation was statistically significant. D,E) GSEA and heatmap of gene set involved in HIF‐1 signal pathway. F) Protein‐protein interaction network analysis of selected molecules. G) The binding site in the promoter area for HIF‐1α transcription. H,I) ChIP and dual‐luciferase reporter assays were performed to confirm the regulatory effects of XBP1 on HIF‐1α expression (*n* = 3). J) Molecular docking model of KD interacting with IRE1α. K) The interaction between KD and IRE1α was assessed by pull‐down assay (*n* = 3). L) Co‐localization of KD and IRE1α was visualized via immunofluorescence staining (*n* = 3). M) CETSA was carried out to evaluate the thermal stabilization of IRE1α protein across varying temperatures (*n* = 3). N) Drug affinity responsive target stability (DARTS) was performed to show the protective roles of KD binding on IRE1α protein against pronase digestion (*n* = 3). O) The expression of XBP1, HIF‐1α, LDHA, PKM2 and HK2 in BMDMs with different treatments (*n* = 3). P) Seahorse assay was used to measure the value of ECAR (*n* = 3). Q) Levels of pro‐inflammatory signaling factors and cytokines were detected using western blot (*n* = 3). Data were presented as mean ± SD. Statistical analysis was performed using one‐way ANOVA followed by Tukey's post hoc test. *p < 0.05, **p < 0.01, ***p < 0.001. NS: Not Significant.

Subsequently, RNA sequencing analysis was performed on TMAO‐induced macrophages following the treatment of KD to ascertain the drug‐related potential molecular targets. In total, 232 differentially expressed genes (DEGs) were acquired, of which 101 were up‐regulated and 131 were down‐regulated in the TMAO + KD group (KD) in contrast to those of the TMAO group (Con), as indicated by the volcano plot (**Figure** **3**A). Gene set enrichment analysis (GSEA) performance manifested that KD management inhibited the expression of gene clusters implicated in inflammation activation (Figure S4A–D, Supporting Information). In addition, findings of GSEA revealed that KD repressed glycolysis pathway (NOM p‐value < 0.01, FDR q‐value = 0.248) and activated mitochondrial fatty acid β oxidation cascade ((NOM p‐value < 0.01, FDR q‐value = 0.203), separately (Figure 3B,C). More importantly, we discovered that KD administration effectively suppressed the expression of transcriptional factor hypoxia inducible factor‐1α (HIF‐1α) and its downstream target molecules, especially glycolysis‐related rate‐limiting enzymes (Figure 3D,E; Figure S5A,B, Supporting Information), demonstrating the crucial roles of HIF‐1α involved in KD‐triggered glycolysis activity regulation. Then, via constructing the protein‐protein interaction network diagram using the STRING database, X‐box binding protein 1 (XBP1) was identified as a potential signal regulator participating in the biological processes of HIF‐1α (Figure 3F). In addition, our findings indicated that silencing of XBP1 markedly weakened the expression of HIF‐1α whereas overexpression of XBP1 obviously elevated the level of HIF‐1α, which had been corroborated by western blot and qRT‐PCR analysis (Figure S6A–C, Supporting Information). These results, along with the previous studies, implied that XBP1 acted as the upstream factor which transcriptional mediated the biosynthesis of HIF‐1α. To verify this hypothesis, the online tool JASPAR (http://jaspar.elixir.no/) was utilized and a putative motif for XBP1 binding site (BS) was disclosed within the HIF‐1α promoter (Figure 3G). Subsequently, ChIP‐qPCR was employed to validate the predicted site in the promoter region of HIF‐1α. The findings of Figure 3H showed that XBP1 directly bound to the response element (RE) and the enrichment of anti‐XBP1 antibody in the binding site was inhibited by KD or si‐XBP1 incubation. Then, the mutagenesis in the binding site was conducted, accompanied by the execution of dual luciferase reporter examination. Consistently, the increased luciferase activity in the overexpression of XBP1 (OE‐XBP1) group was abolished after KD administration, and observed luciferin level in the siRNA‐ XBP1 (si‐XBP1) group was reduced in contrast to that of the control group. Meanwhile, the enhanced effect of XBP1 overexpression and impaired effect of XBP1 gene silencing on regulating HIF‐1α promoter activity were both disappeared as long as the emergence of mutating RE site (Figure 3I).

There are lines in the literature illustrating that inositol‐requiring enzyme 1α (IRE1α)/XBP1 pathway exerts inextricable roles in the onset of endoplasmic reticulum (ER) stress.^[^ ^29^ ^]^ Chronic exposure to persistent stress conditions, redundant misfolded proteins are produced and accumulate to induce unfolded protein responses which are mediated by ER sensors like IRE1α. Once activated, IRE1α acquires ribonuclease capacities and then excises the intron in XBP1 mRNA, which is linked to the transcriptional activity maturation of XBP1.^[^ ^30^, ^31^ ^]^ Since our results revealed that KD treatment decreased the protein content of XBP1, without affecting its mRNA level in TMAO‐insulted macrophages (Figure S5C,D, Supporting Information), we postulated that KD‐induced translation repression of XBP1 was associated with the activation inhibition of IRE1α. As expected, we discovered that KD administration abolished the activity of IRE1α, as demonstrated by the decrement of protein phosphorylation level (Figure S7A, Supporting Information). Additionally, GSEA assessment showed that ER‐related protein processing was weakened by KD treatment, which might reflect alleviation of ER stress via targeting IRE1α (Figure S7B, Supporting Information). Next, molecular docking was adopted to validate whether KD directly interacted with the active site of IRE1α to repress its activation. Base on the results of Autodock Vina, a binding mode between KD and IRE1α was successfully implemented and visualized using PyMol, predicting that KD probably formed hydrogen bond link with the amino acid residue LYS‐599 and GLU‐612 of IRE1α, with an approximate free energy binding of ‐6.2 kcal mol^−1^ producing strong binding stability (Figure 3J).

Then, biotin‐labeled KD (biotin‐KD) was synthesized and a biotinylated‐protein interaction pull‐down test displayed that biotin‐KD probe effectively combined with IRE1α in lysates from BMDMs (Figure 3K). Immunofluorescence staining also showed the colocalization of biotin‐KD with IRE1α, which was consistent with that gained from the pull‐down assay (Figure 3L). Via evaluating changes in the thermal stability of proteins, cell thermal shift assay (CETSA) was applied to determine ligand‐protein engagement. As described in Figure 3M, KD treatment dramatically strengthened the thermal stability of IRE1α, as observed by attenuation of temperature‐dependent denaturation, compared to the control group. Moreover, we discovered that KD concentration‐dependently confined pronase‐induced degradation of IRE1α, further supporting the superior bioconjugation capability of KD with IRE1α (Figure 3N).

To further verify the implication of KD in the mediation of IRE1α/XBP1/HIF‐1α signal cascade and downstream glycolytic processes, gene expression and enzyme activity regulation were performed. Following the management of 4μ8C serving as antagonist of IRE1α ribonuclease, the level of XBP1 and HIF‐1α was comparable to that of KD treatment and markedly lower than that of TMAO incubation alone (Figure 3O). In addition, no significant changes in the expression of HK2, PKM2 and LDHA, glucose consumption, lactate concentration and ECAR level had been noticed for the KD and 4μ8C group. Moreover, overexpression of HIF‐1α (OE‐HIF‐1α) failed to rescue depressed activity of IRE1α‐XBP1 axis caused by KD, whereas enhanced the glycolytic activities (Figure 3O,P; Figure S8A–C, Supporting Information). Cohesively, we found that KD‐aroused HIF‐1α downregulation, glucose utilization diminution, reduction of lactate release, and ECAR value were counteracted by the IRE1α agonist IXA4, which, in turn, was repressed by siRNA‐HIF‐1α (si‐HIF‐1α) incubation, as exemplified by the results of Figure 3O,P and Figure S8C (Supporting Information). Then, the association of KD‐induced XBP1/HIF‐1α glycolytic pathway suppression with inflammation improvement was evaluated. We discovered that KD and 4μ8C both restrained STAT1 and NF‐κB pathway activation and encumbered the expression of M1 factor iNOS, IL‐6, TNF‐α and MCP‐1 both at the transcriptional and translational level in TMAO‐stimulated BMDMs, which was abrogated by OE‐HIF‐1α administration. Moreover, intracellular inflammation responses were revived when exposed to IXA4 and were prohibited again after si‐HIF‐1α addition (Figure 3Q; Figure S9A–D, Supporting Information). These findings thereby potentiated the understanding of KD‐triggered inflammation alleviation via decreasing glycolysis activities relying on IRE1α/XBP1/HIF‐1α cascade.

Intriguingly, we observed that KD‐induced activation of anti‐inflammatory axis STAT6 could be imitated by 4μ8C and counteracted by IXA4 separately, while which was not affected by HIF‐1α expression change. Meanwhile, the content alteration of M2 marker TGF‐β, IL‐10, IL‐4 and Arg‐1 presented the similar trend (Figure S9A–D, Supporting Information). These results suggested that the enhanced effected produced by KD on anti‐inflammatory pathway activity was derived from ER stress repression but not from downstream glycolysis inhibition. Then GSEA revealed that KD treatment significantly enhanced the activity of Parkin‐dependent mitophagy pathway and western blot detection discovered that the level of XBP1, instead of HIF‐1α, was negatively correlated with the activation of mitophagy cascade (Figure S10A,B, Supporting Information). Since repressed mitophagy is associated with mitochondrial homeostasis dysfunction and abnormality of OXPHOS and ER stress is deemed as a predisposing factor driving the disorder of mitochondrial quality control, it is possible that blockade of ER stress by KD is sufficient to normalize the mitophagy process and improve mitochondrial homeostasis and then facilitate the TCA cycle.^[^ ^32^, ^33^ ^]^ Indeed, our findings manifested that both of KD and 4μ8C restored mitochondrial membrane potential, accompanied by up‐regulation of IDH1 and elevation of α‐KG production. Consistent with the variation tendency of intracellular anti‐inflammatory factors, decrease of membrane potential and reduction of IDH1 content, along with the level decline of α‐KG, were seen in KD‐treated BMDMs after IXA4 addition, which was unaffected following HIF‐1α overexpression or silencing intervention (Figure S8A,D,E; Figure S10C, Supporting Information). As was reported that OXPHOS promotes M2 phenotype formation of macrophages,^[^ ^27^ ^]^ KD might improve internal environment of mitochondria to maintain the level of functional proteins, thereby enhancing OXPHOS and activating anti‐inflammatory pathways sequentially, which is worth to be further elucidated.

### 2.4 Polarization Reprogramming of Macrophages Triggered by KD Facilitated Angiogenic Processes

**Figure 4:**
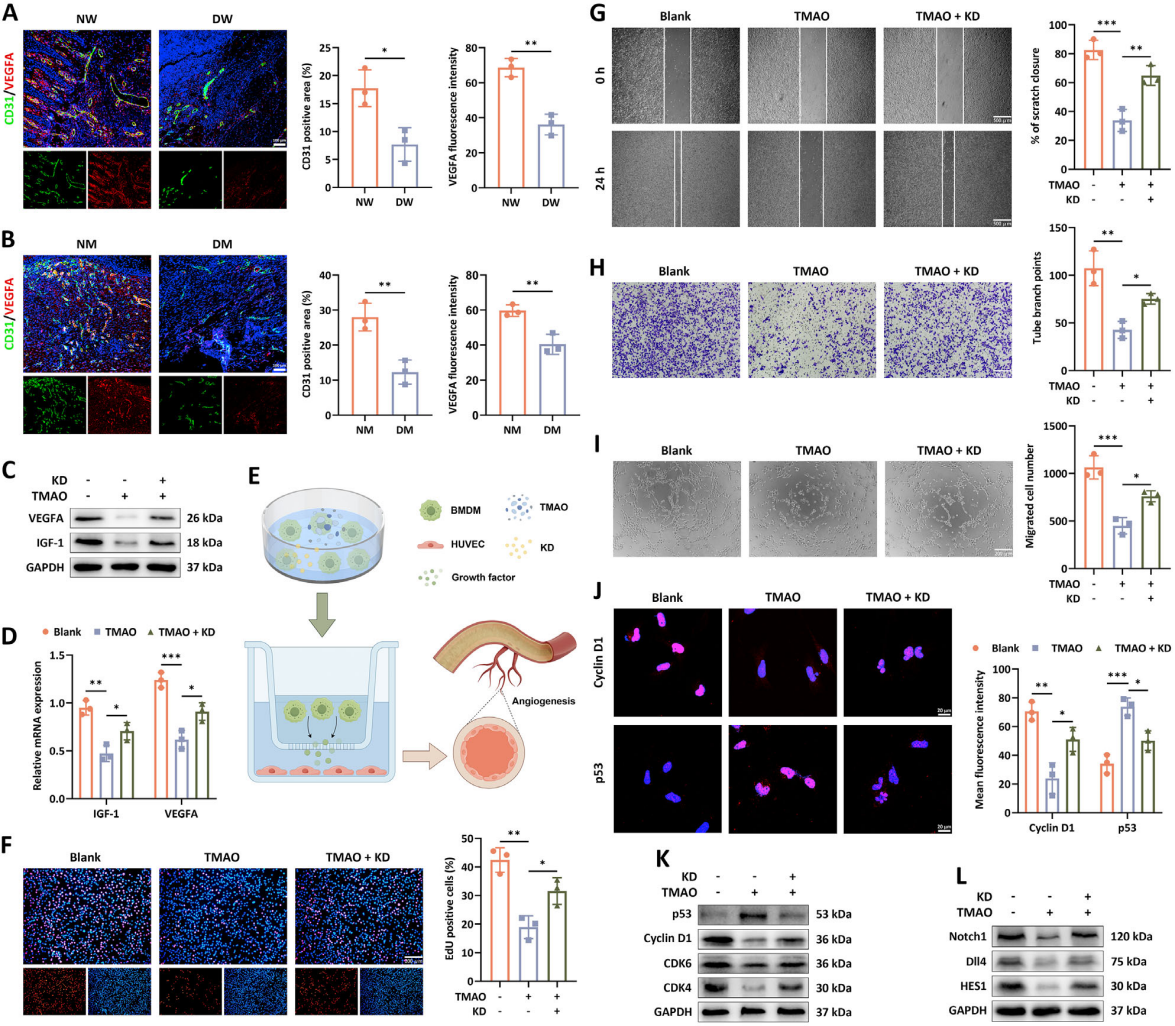
Effects produced by KD‐treated macrophages on the angiogenic processes. A,B) Immunofluorescence staining revealed the expression of CD31 and VEGFA in the normal wound tissue and diabetic wound area from patients and mice, respectively (*n* = 3). C,D) The protein and mRNA contents of VEGFA and IGF‐1 in BMDMs (*n* = 3). E) The diagram showed the effects of primed BMDMs on the angiogenic abilities of HUVECs. F) Proliferative property of HUVECs after relevant treatments measured by EdU assay (*n* = 3). G) Cell scratch assay and H) transwell assay was employed to ascertain the migratory ability of HUVECs (*n* = 3). I) Representative images of tube formation assay (*n* = 3). J) Immunofluorescence analysis and K) western blot was carried out to determine the expression of cell cycle‐related signal proteins (*n* = 3). L) Contents of Notch1, Dll4 and HES1 in HUVECs with varying interventions (*n* = 3). Data were presented as mean ± SD. Statistical significance was determined using Student's t test for A,B, one‐way ANOVA followed by Tukey's post hoc test for C,D,F‐L. *p < 0.05, **p < 0.01, ***p < 0.001.

Angiogenesis, a tightly controlled process, is featured by vascular endothelial cells proliferate and migrate to arrange into lumen of newly formed capillaries sprouting from preexisting vessels supporting nutrients supply.^[^ ^34^ ^]^ Violent inflammatory reactions developed in diabetic wounds are capable of slowing down tissue healing by disrupting pro‐angiogenic events.^[^ ^35^ ^]^ In consistent with previous studies, there was a decreased vascular density and lowered expression of pro‐proliferative cytokine in the diabetic wound in contrast to that in normal wound area, as assessed by immunofluorescence staining of CD31 and VEGFA (**Figure** **4**A,B). Given that timely phenotypic transition from M1 to M2 not only restrained inflammatory injury but also facilitated growth factors release to promote tissue regeneration, we assessed the pro‐proliferative potential of BMDMs under the stimulation of TMAO and the intervention effects of KD. Our findings showed that TMAO significantly decreased the expression of VEGFA and insulin‐like growth factor‐1 (IGF‐1) in macrophages, whereas these events were reversed following KD treatment (Figure 4C,D). Then we probed whether the microenvironment containing TMAO‐insulted macrophages with or without KD intervention affected the biological functions involving angiogenesis of endothelial cells (Figure 4E). As shown in Figure 4F, the ratio of EdU+ HUVECs co‐cultured with BMDMs pretreated with TMAO was obviously reduced, yet the proportion of EdU+ cells was increased after KD addition to the TMAO niche. Results from the wound scratch and transwell migration assay indicated that the crosstalk with inflammatory macrophages significantly restrained the mobilization ability of HUVECs. KD‐treated BMDMs markedly accelerated the rate of cell scratch closure and enhanced HUVECs mobilization to the lower chamber when compared to the TMAO pre‐stimulation alone (Figure 4G,H). Similarly, we observed that the number of tube branch points formed by HUVECs was decreased when co‐cultured with TMAO‐induced macrophages, while the tube formation ability of HUVECs was significantly enhanced in the KD‐affected group (Figure 4I). In addition, KD normalized the reduced expression of cyclin D1, cyclin‐dependent kinase 4 (CDK4) and CDK6 participating in cell cycle transition and the elevated level of p53 responsible for cell cycle arrest in HUVECs cocultured with TMAO‐provoked macrophages, implying the importance of proliferation facilitation in pro‐angiogenic mechanisms of this phytochemical (Figure 4J,K). Notch pathway, an evolutionary conserved signaling cascade, is documented to possess critical roles in guiding embryonic development, tissue repair and vessel sprouting.^[^ ^36^ ^]^ Subsequently, with the western blot test, our results presented a decreased trend on the expression of delta like canonical Notch ligand 4 (Dll4), Notch1 and hes family bHLH transcription factor 1 (HES1) in HUVECs after neighboring TMAO‐irritated BMDMs, which then was ameliorated by KD application (Figure 4L). Hence, these findings proposed that KD‐tilted M2 phenotype of macrophages induced the release of adequate bioactive cytokines, the latter enhanced activities of Notch signal pathway governing angiogenesis process and accelerating tissue repair.

### 2.5 Preparation and Characterization of the Hydrogel

**Figure 5:**
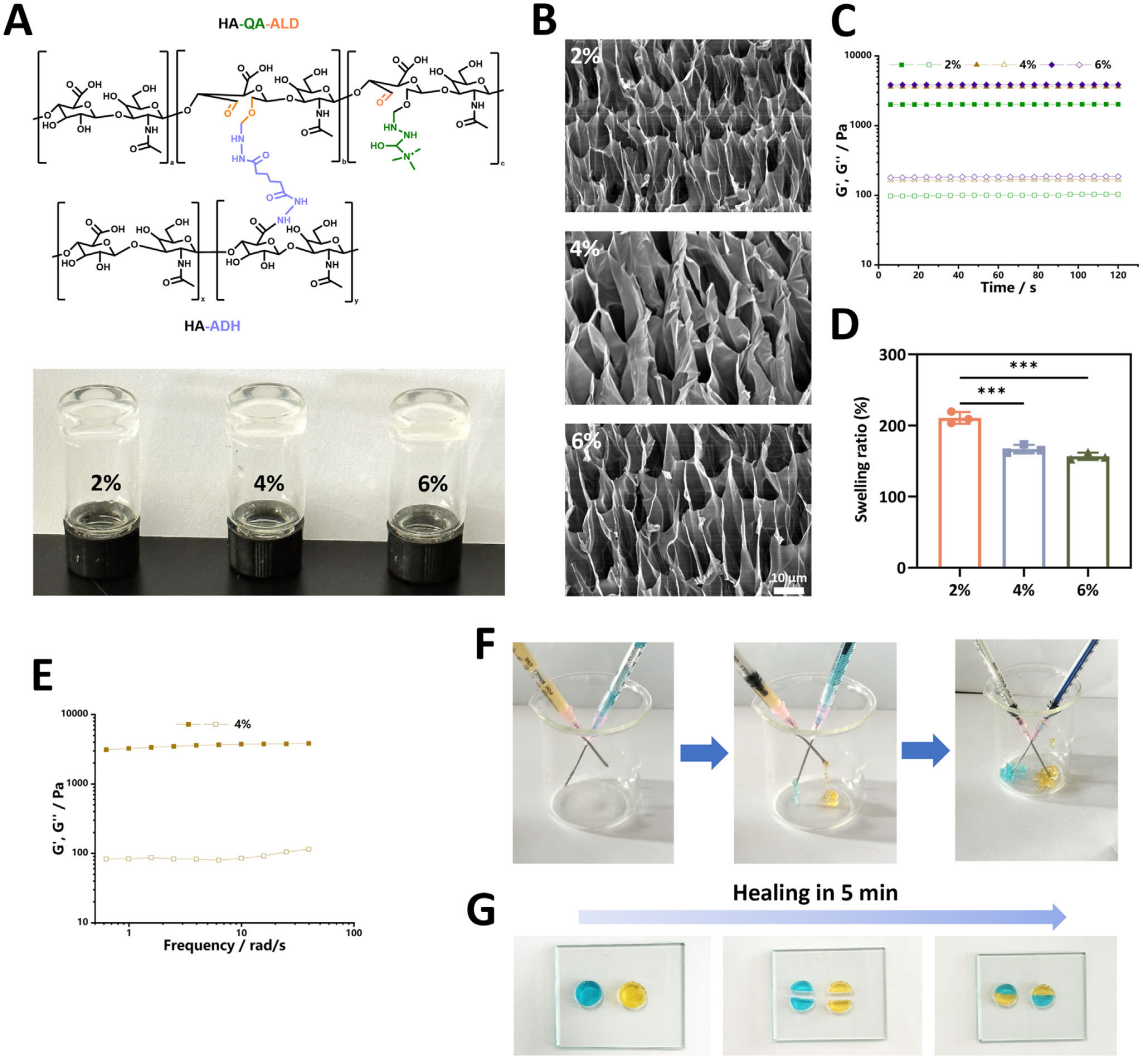
Preparation and characterization of the HAQA hydrogel. A) Structural formula for the cross‐linking of HA‐QA‐ALD and HA‐ADH polymers and the digital photographs of the hydrogel. B) Scanning electron microscope images of the HAQA hydrogel microstructure. C) The rheological time sweep sweep test of HAQA hydrogel. Solid symbols marked storage modulus (G’) and hollow symbols marked loss modulus (G’’). D) Swelling ratio of hydrogel in PBS buffer for 10 h. E) The frequency sweep test of HAQA hydrogel. F) Injectability of the hydrogel, where yellow and blue dyes were added into hydrogel for the convenience of observation. G) The self‐healing properties of hydrogels could be demonstrated by the integration of different colors after incision. Data were presented as mean ± SD. Statistical analysis was performed using one‐way ANOVA followed by Tukey's post hoc test. ***p < 0.001.

To better deliver KD to the depths of the diabetic wounds, we designed a modified hyaluronic acid (HA) MN with good antibacterial and antioxidative properties. First, we synthesized two kinds of HA derivates, HA modified with quaternary ammonium groups and aldehyde groups (HA‐QA‐ALD) and HA modified with hydrazide groups (HA‐ADH) (**Figure** **5**A; Figure S11, Supporting Information). By simple equal volume mixing, a well crosslinked HAQA hydrogel could form quickly under different final solid concentration by the Schiff Base Reaction (2%, 4%, and 6%, Figure 5A). The scanning electron microscope images showed that all these three groups had obvious 3D connected porous structure (Figure 5B).

Then, we tested the viscoelasticity of this HAQA hydrogel by rheological test. The time sweep results demonstrated that with the increasing of the solid concentration, the modulus of HAQA hydrogel also increased. When the solid concentration reached 4%, the increasing tendency of modulus would slow down (Figure 5C). The following swelling ratio histogram was also consistent with this rheological data. With the increased solid concentration, the swelling ratio significantly decreased, due to the increased crosslinking density (Figure 5D). Then, we chose 4% group as the representative for the frequency sweep. The modulus of this HAQA hydrogel showed slightly frequency‐dependent characteristic (Figure 5E), which proved the dynamic crosslinking property of HA hydrogel. Based on the dynamic property, this HAQA hydrogel showed outstanding injectable property after gelation (Figure 5F; Video S1, Supporting Information), which guaranteed the effective shape forming of our microneedle after KD loading. Moreover, this HAQA hydrogel also performed good self‐healing property as shown in Figure 5G and Video S2 (Supporting Information), because of the dynamic formation and unraveling of the hydrazone crosslinks. We believed that this self‐healing property accompanied by the crosslinked design was vital for our designed MN to maintain their integrity with a longer time after in vivo implantation than the common uncrosslinked pure HA microneedle. Through the analysis of these properties of the HAQA hydrogel, we decided to selected 4% group with a moderate modulus and swelling ratio to prepare the KD encapsulated MN for the following in vitro and in vivo researches.

### 2.6 Mechanical and Functional Properties of the M‐KD@HAQA‐MN Patch

**Figure 6:**
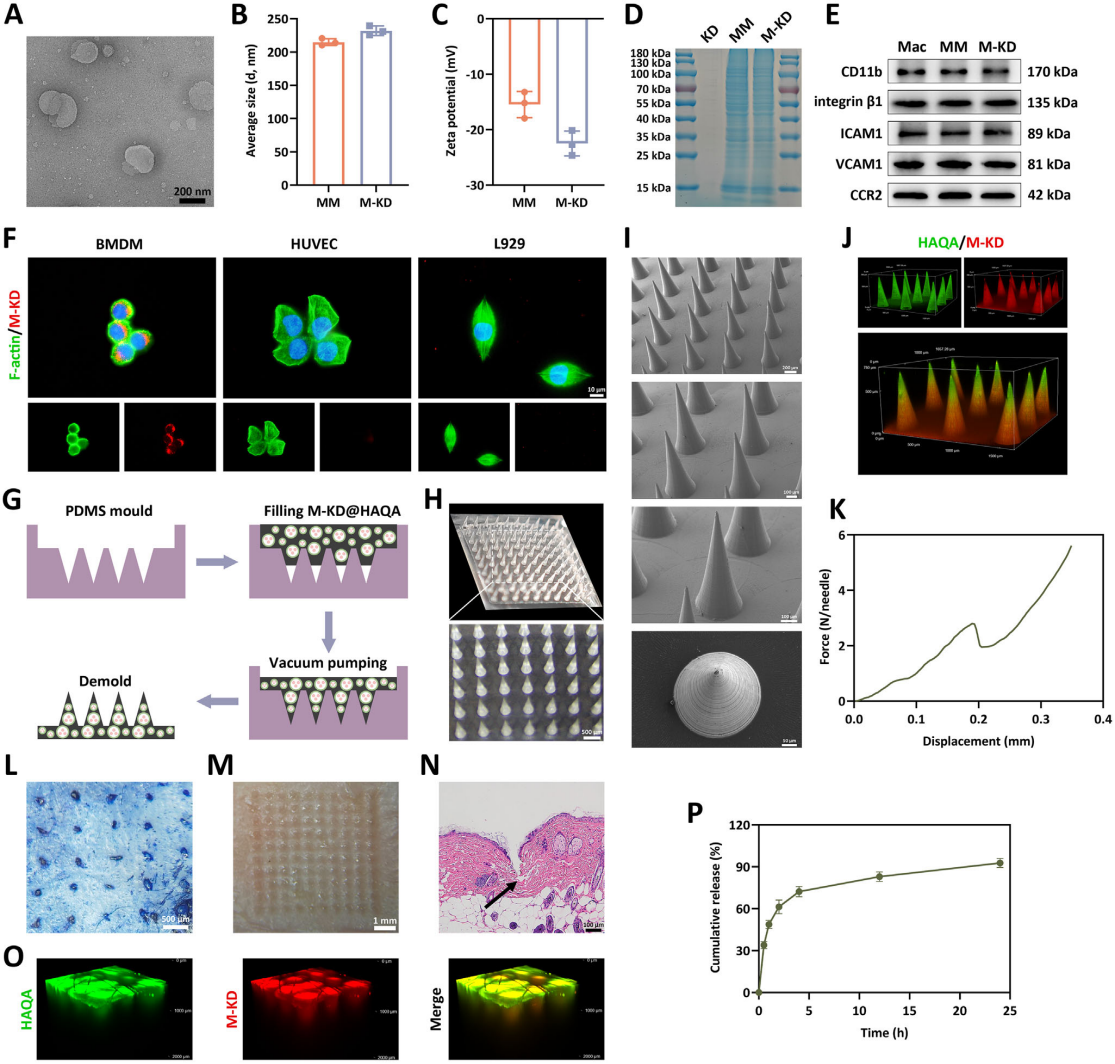
Fabrication of characterization of M‐KD@HAQA‐MN. A) The morphology of membrane‐coated KD (M‐KD) captured by a transmission electron microscope. B) Hydrodynamic diameter and C) zeta potential of the macrophage membrane (MM) and M‐KD (*n* = 3). D) Coomassie brilliant blue staining was employed to evaluate the protein profiles of MM and M‐KD, KD was set as a control group (*n* = 3). E) The expression of CD11b, integrin β1, ICAM1, VCAM1 and CCR2 in the lysates from macrophages (Mac), MM and M‐KD, respectively (*n* = 3). F) the Cy3‐labelled M‐KD (red fluorescence) inside the FITC‐labelled cytoskeleton (green fluorescence) was visualized by the confocal laser scanning microscopy (*n* = 3). G) Fabrication procedure of M‐KD@HAQA‐MN. H,I) The appearance of the MN patch under a stereomicroscope and scanning electron microscope. J) Confocal microscopy image of HAQA‐MN (red) loading M‐KD (green). K) Force‐displacement curve from compression of the M‐KD@HAQA‐MN patch (*n* = 3). L) Penetration of the MN patch into the murine and M) porcine dorsal skin (*n* =3). N) H&E staining and O) confocal microscopy images of a murine back skin inserted by the M‐KD@HAQA‐MN (*n* = 3). P) Release curve of KD from the MN system in vitro (*n* = 3). Data were presented as mean ± SD.

Due to insufficient availability of phytochemicals when utilized alone, macrophage membrane‐coated KD was designed to achieve the purpose of affinity enhancement and escape from the elimination by immune system. The morphology of M‐KD was visualized by transmission electron microscopy, which showed a homogeneous hollow spherical structure (**Figure** **6**A). Dynamic light scattering detection indicated that M‐KD had a hydrodynamic diameter increased by ∼15 nm than that of macrophage vesicle (Figure 6B). Moreover, the zeta potential of the vesicle underwent reduction after the deposition of KD, suggesting that the drug was successful encapsulated by the macrophage membrane (Figure 6C). Then sodium dodecyl sulfate‐polyacrylamide gel electrophoresis (SDS‐PAGE) was carried out and Coomassie brilliant blue staining demonstrated that M‐KD inherit the majority of protein profiles from the macrophage membrane (Figure 6D). Noteworthy, as was analyzed by western blot, the biomarkers including CD11b, integrin β1, intercellular cell adhesion molecule‐1 (ICAM1), vascular cell adhesion molecule 1 (VCAM1) and C‐C motif chemokine receptor 2 (CCR2) committed to recognize and adhere were enriched and well‐preserved following the process of isolation and encapsulation, further validating functionalized integrity of M‐KD alike to the macrophage membrane (Figure 6E). In addition, the drug loading efficiency of M‐KD was calculated to be ≈15.52%. To confirm whether membrane coating endowed KD with high affinity toward macrophages, M‐KD was labelled with Cy3 and laser scanning confocal microscope measurement was conducted. As anticipated, our results revealed that M‐KD entrance into the BMDMs displayed a considerably increased content in comparison with those of HUVECs and L929 fibroblasts (Figure 6F; Figure S12A, Supporting Information).

In view of the rapid release, low retention and poor availability of drugs during in vivo application,^[^ ^37^ ^]^ HAQA hydrogel‐based microneedle loading M‐KD delivery system was developed. The M‐KD@HAQA‐MN patch was fabricated by a mold template casting procedure (Figure 6G). Morphological evaluation disclosed the intact tip shape and sharp point of each needle, orderly aligned on the backing layer in a 10×10 array, with a needle height, base width and tip‐to‐tip spacing of 800, 300 and 650 µm respectively (Figure 6H,I). 3D reconstructive fluorescence images uncovered that Cy3‐probed M‐KD was uniformly distributed in the hydrogel MN (Figure 6J). Next, mechanical strength of obtained MN patch was determined and the force curve showed that the fracture force exceeded 2 N/needle, signifying outstanding mechanical characters sufficient to pass through skin barrier without breaking (Figure 6K).^[^ ^38^, ^39^, ^40^ ^]^ Indeed, an array of holes and traces stained with trypan blue was evident appeared in murine back skin following insertion of the MN patch (Figure 6L). This penetrative ability had been corroborated by the penetration test performed on porcine skin (Figure 6M). On account of the sharp conical structure and suitable height of each needle, the delivery vehicle was qualified to pierce through the stratum corneum and enter the dermis, as testified by the histological examination (Figure 6N). In parallel, Figure 6O uncovered that the unique morphology of the MN enabled M‐KD to reach different depths of skin tissue, then conquering the drawback of limited target regions of conventional hydrogel dressings. With respect to the drug release property, we observed that cumulative release of KD from the MN system was gradually increased, reaching ≈50% and 1 h and ≈ 90% at 24 h, in contrast to burst release of KD dissolved in PBS (Figure 6P). Additionally, the in vivo release curve showed that less than 50% content of the drug was discharged at 8 h, ≈ 80% at 32 h and then nearly 90% at 48 h, further highlighting the controlled release kinetics (Figure S12B, Supporting Information).

Because of the susceptibility of hyperglycemic condition to microbial invasion, bacterial infection is not uncommon in diabetic wound areas and exacerbated tissue damage via disrupting oxygen and nutrient supply.^[^ ^41^ ^]^ Herein, *E. coil* and *S. aureus* were selected as the bacterial strains to assess the anti‐bacterial ability of the MN array in vitro. Quantitatively analysis of the plate counting method revealed that HAQA‐MN and M‐KD@HAQA‐MN shared similar abilities hindering the proliferation of *E. coil* (Figure S13A, Supporting Information). Compared to the control group, a *S. aureus* scavenging effect was exposed after HAQA‐MN treatment, and which was not affected by M‐KD addition (Figure S13B, Supporting Information). The results of live/dead assay agreed with the plate counting, showing that the red fluorescence intensity (dead bacteria) was significantly enhanced after the treatment of MN patch, irrespective of the presence of M‐KD (Figure S13C,D, Supporting Information). The positive charge of QA radical in the HA‐QA‐ALD hydrogel is capable of disrupting the negative charge‐carrying bacterial cell wall via the electrostatic interaction, leading to the outflow of internal contents and death of the bacteria, which might be the anti‐bacterial mechanism of the HA‐ADH/HA‐QA‐ALD hydrogel. Despite that low content of ROS is beneficial for pathogens clearance, excessive ROS are able to induce cellular death and amplify inflammation extent for discouraging wound healing.^[^ ^42^ ^]^ As was reported before, our results observed that ROS level of diabetic wounds was significantly higher than that of normal wounds from both of the patients and mice models (Figure S14A,B, Supporting Information). To ascertain the roles of M‐KD@HAQA‐MN in eliminating intracellular ROS, the co‐culture model and DCFH‐DA probe was employed. We observed that whether M‐KD was loaded, HAQA‐MN patch dramatically decreased ROS content in H2O2‐stimulated HUVECs. Consistently, the increased level of ROS in L929 cells induced by H2O2 was abolished following HAQA‐MN intervention with or without M‐KD deposition (Figure S14C, Supporting Information). In addition, for determining the anti‐oxidative mechanism of the hydrogel MN, we detected the ROS‐scavenging factors including NQO‐1 and CAT in the HUVEC and L929 cells (Figure S14D,E, Supporting Information). We observed that the expressions of these anti‐oxidative factors were significantly increased following the treatment of the MN patch, which was the possible mechanism by which the HA‐ADH/HA‐QA‐ALD hydrogel decreased the level of ROS. Subsequently, using the live/dead staining reagents, we found that M‐KD@ HAQA‐MN not only unaffected the viabilities of HUVECs and L929 cells but also protected them from oxidative injury, affirming the biocompatibility and pro‐survival capacities of the drug delivery system (Figure S14F,G, Supporting Information).

### 2.7 M‐KD@HAQA‐MN Promoted Cutaneous Wound Healing in Diabetic Mice

**Figure 7:**
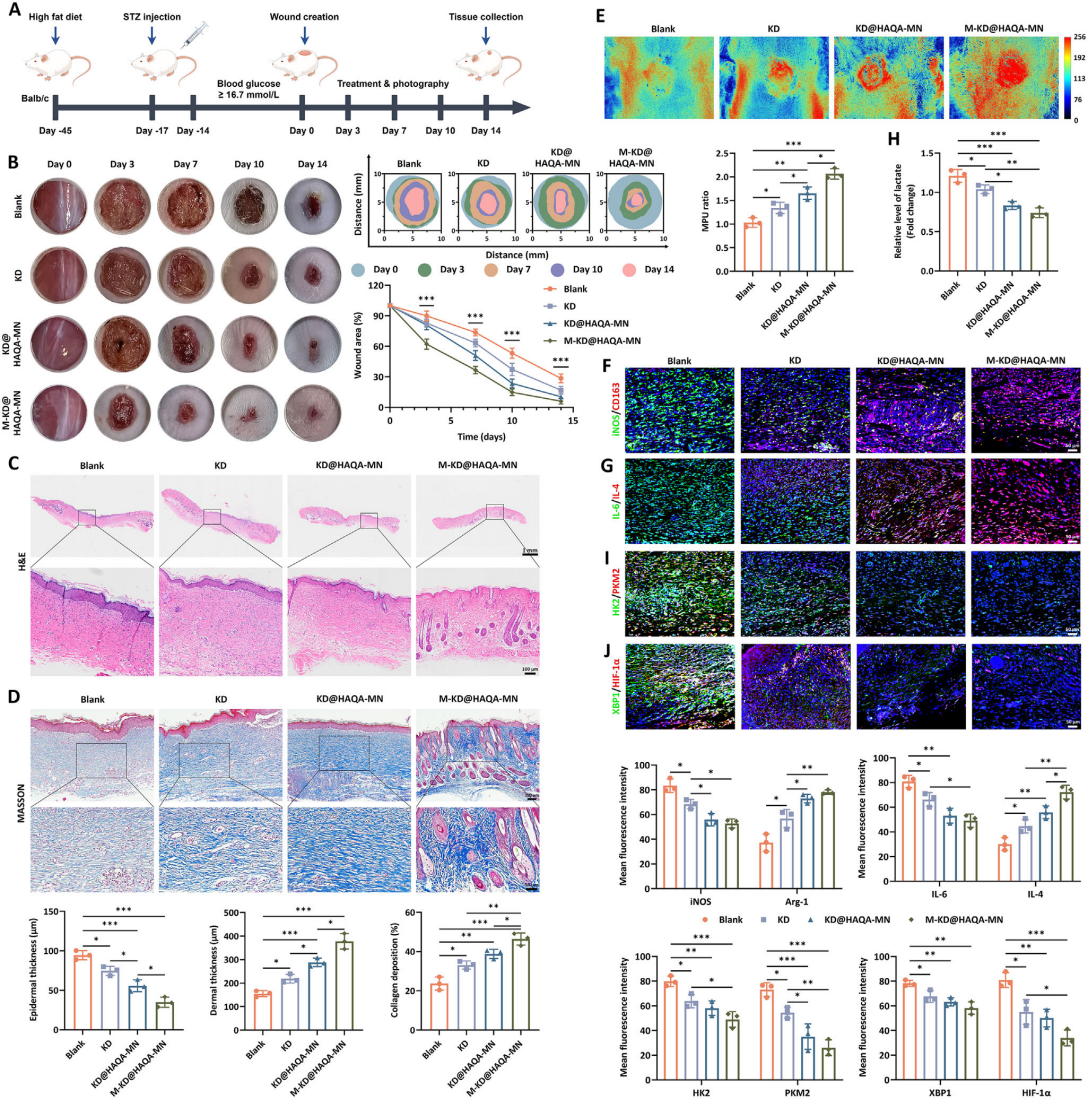
Treatment of the M‐KD@HAQA‐MN delivery system promoted diabetic wound healing of mice. A) Diabetic wound establishment and treatment procedure. B) Images of wound healing processes (*n* = 4). C) H&E and D) MASSON staining of wound tissues with indicated treatments (*n* = 3). E) The blood flow of wound area was measured using the laser speckle contrast imaging system (*n* = 3). F,G) The expression of M1 marker iNOS and IL‐6 and M2 marker of CD163 and IL‐4 in the wound tissues (*n* = 3). H) Relative level of lactate of wound tissues with varying treatments (*n* = 3). I,J) Immunofluorescence analysis of HK2, PKM2, XBP1 and HIF‐1α in the wound tissues (*n* = 3). Data were presented as mean ± SD. Statistical analysis was performed using one‐way ANOVA followed by Tukey's post hoc test. *p < 0.05, **p < 0.01, ***p < 0.001.

Building upon the findings that M‐KD@HAQA‐MN displayed multifunctional behaviors against inflammation progression, bacterial infection and oxidative stress, we then investigated its involvement in the wound repair process of diabetic mice. With the combination of high fat diet and intraperitoneal injection of streptozocin (STZ), murine model of diabetes was established, accompanied by the creation of full‐thickness cutaneous defect wound (≈ 10 mm in diameter) on the back. Afterwards, intervention approaches were performed varying according to different groups and macroscopic views reflecting repair development of skin wound were obtained at days 0, 3, 7, 10 and 14 post‐operation (**Figure** **7**A). As depicted in Figure 7B, there was an obvious difference existed in the healing rate among four groups. The residual wound areas of the Blank, KD and KD@HAQA‐MN groups reduced successively, presenting closure rates of 26.38%, 36.74%, and 49.05% on day 7 and 71.47%, 83.12% and 89.51% on day 14, separately. In contrast, wounds treated with M‐KD@HAQA‐MN exhibited the fastest healing rate at each time point, with an almost completely healed state at the experimental endpoint (closure rates ≈ 93.9%), yet incomplete skin barrier was apparently seen in the other groups.

Histological features during the course of cutaneous tissue repair consist of re‐epithelialization, collagen deposition, granulation formation, blood flow increase, dermis thickness and hair follicle production.^[^ ^43^ ^]^ In this study, the findings of histopathologic measurement were in consistent with the macroscopic observation. Hematoxylin and eosin (H&E) staining discovered that KD‐treated groups exhibited thinner epithelial layer than the Blank group, among which epithelial thickness of KD exceeded that of KD@HAQA‐MN group and skin tissues affected by M‐KD@HAQA‐MN possessed the thinnest epidermis than others, which was similar to the normal epithelial layer, hinting that scar hyperplasia was inhibited after KD administration without affecting re‐epithelialization.^[^ ^44^ ^]^ In contrast, the dermal thickness was increased in the order of Blank < KD < KD@HAQA‐MN < M‐KD@HAQA‐MN group, as supported by the data of Figure 7C. In addition, Masson's trichrome staining indicated an orderly arranged collagen with the volume fraction scaling up to ≈46% in M‐KD@HAQA‐MN‐triggered wounds, which was apparently higher than KD@HAQA‐MN and KD‐treated skin tissues, not to mention tissue samples in the Blank group (Figure 7D). Using the laser speckle imaging technology, we found that KD administration yielded enhanced skin blood flow, accompanied by better blood perfusion as long as KD@HAQA‐MN was applied, while which was still inferior to the treatment of M‐KD encapsulated in the MN (Figure 7E). Hence, these data confirmed the contributing roles of KD in diabetic wound healing and its therapeutic actions enhanced by the MN delivery vehicle for favoring skin tissue regeneration, collagen deposition and blood supply.

Considering the potent effects of KD on suppressing macrophage inflammation, we assessed the inflammatory profiles in the wound tissue treated with different strategies. Immunostaining detection of macrophage polarization markers suggested that the level of iNOS in the skin tissue treated by KD was higher than those of KD@HAQA‐MN and M‐KD@HAQA‐MN intervention and lower than that of the Blank, whereas the expression of CD163 displayed opposite results, corroborating with the foregoing in vitro data regarding the M2 polarization incline of KD (Figure 7F). Additionally, low levels of IL‐6, TNF‐α and MCP‐1 and high contents of IL‐4, TGF‐β and IL‐10 were observed in KD‐treated wounds and the difference was increasing gradually as KD processing changed (Figure 7G; Figure S15A,B, Supporting Information). Similar trends had also been found in the analysis of pathway activity of NF‐κB, STAT1 and STAT6, which further emphasized the anti‐inflammatory abilities of this phytochemical (Figure S15C, Supporting Information). Next, we investigated the glucose metabolism of wound tissues and discovered that approaches involving KD yielded reduced content of lactate as well as elevated level of α‐KG, followed by down‐regulation of HK2 and PKM2 and up‐regulation of IDH1 (Figure 7H,I; Figure S16A,B, Supporting Information). Then, ER stress‐mediated HIF‐1α pathway was measured and our findings showed that with preparation alterations of KD prior to administration, there as a declining tendency on the activation of IRE1α/XBP1/HIF‐1α signal axis, as proved by results of fluorescence intensity (Figure 7J). Meanwhile, the signaling cascades implicated in mitophagy were also determined. Findings from our study indicated that the activity of PTEN induced putative kinase 1 (PINK1)‐Parkin‐related autophagic pathway was increased in KD‐treated groups (Figure S16C,D, Supporting Information). Similarly, the change magnitude of proteins discussed here mainly depended on the fabrication mode of KD before the application. The above results clarified KD‐induced metabolic reprogramming via regulating ER stress‐related glycolysis and mitophagy‐associated OXPHOS from the perspective of animal models and highlighted the importance of the targeting behavior in strengthening the efficiency of KD.

Moreover, our findings showed that faster wound healing was seen by KD@HAQA‐MN and M‐KD@HAQA‐MN treatment in comparison with the blank and KD group, indicating that antibacterial properties of the hydrogel MN vehicle made up for the drug's lack of repair promotion on infected diabetic wounds (Figure S17, Supporting Information). To further verify the anti‐oxidative property of the MN system in vivo, the DHE probe was employed. As reported in Figure S18A (Supporting Information), KD@HAQA‐MN and M‐KD@HAQA‐MN both produced a fluorescence signal weaker than other groups, whereas KD yielded no obvious reduction on ROS level in comparison with the Blank group, elucidating that combination of KD with the MN enabled the drug‐loaded delivery system to possess anti‐inflammatory and ROS‐scavenging abilities together, thereby displaying faster wound healing rate than either used alone.

Since blood perfusion of wound area was improved following KD treatment, the roles of KD in angiogenesis modulation were explored. We found that the density of CD31 acting as biomarker of newly‐formed vessels had been elevated by KD, yet the effects of KD@HAQA‐MN and M‐KD@HAQA‐MN on CD31 positive area were more obvious (Figure S18B, Supporting Information). In addition, the fluorescence signal of proliferating cell nuclear antigen (PCNA) suggested a proliferation‐promoting condition offered by KD. Such an observation is in line with the expression of VEGFA and IGF‐1, presenting an increasingly influence in case of the usage of membrane coating and MN transport (Figure S18C–E, Supporting Information). Cohesively, compared to the Blank group, levels of Dll4, Notch1 and HES1 were increased by KD stimulation and even higher in KD@HAQA‐MN‐treated tissues. While M‐KD@HAQA‐MN was capable of inducing activation of Notch cascade more effectively than others (Figure S18D, Supporting Information). In terms of the biocompatibility of M‐KD@ HAQA‐MN in vivo, our findings showed that no significant changes of hemolytic test, blood routine and biochemical parameters were observed in the comparison of the Blank, KD, KD@HAQA‐MN and M‐KD@HAQA‐MN groups (Figure S19A–C Supporting Information). The histological features of visceral organs of the Blank group were comparable to that of mice affected by KD and also close to MN‐treated mice (Figure S19D, Supporting Information).

## 3 Discussion

A bunch of literature has demonstrated that the high incidence and poor prognosis of diabetic wounds could be explained by the multiple pathogenic factors existed in the hyperglycemic microenvironment, including immoderate inflammation, oxidative stress and bacterial infection, which in turn determine the limited efficiency of therapeutic approaches so far.^[^ ^4^, ^5^, ^6^, ^45^ ^]^ In this study, we introduced a multifunctional drug delivery system with highly effective effects for the management of diabetic wounds, relying on the incorporation of macrophage inflammation inhibition produced by KD‐trigged metabolic reprogramming with the anti‐bacterial and anti‐oxidative abilities of the HAQA‐MN. The development of the HA‐based MN system encapsulated with KD coated within macrophage membrane represents a significant advancement in addressing the multifaceted challenges of diabetic wound repair.

Long‐term exposure to the etiological factors in the local tissue, macrophages are overactivated to continuously generate inflammatory cytokines which retard transition from inflammation to proliferation phase and weaken angiogenic functions of endothelial cells, thus exerting detrimental behaviors against wound healing.^[^ ^46^ ^]^ TMAO acting as a pro‐inflammatory factor not only largely exists in the circulation of diabetic individuals but also be found to accumulate in the diabetic wound area in this study, implying the contributing roles of TMAO in inflammation persistence within the wound tissue, providing deeper understanding on the delayed healing mechanisms associated with the diabetic immune microenvironment.

Several lines of evidences have elaborated the pivotal roles of metabolic patterns in defining the functional phenotypes of immune cells. For instance, the activity of glycolysis is advantageous over the OXPHOS in M1 macrophages, which lead to the aggregation of glycolytic metabolites responsible for the activation of pro‐inflammatory cascades including NF‐κB and STAT1.^[^ ^27^, ^47^ ^]^ Yet anti‐inflammatory M2 macrophages experience the contrary tendency, inducing the production of TCA cycle‐related metabolites, the latter then activate key pathways like STAT6 to impede inflammation progression.^[^ ^48^, ^49^ ^]^ Consequently, agents capable of affecting metabolic reprogramming have been sought as potential candidates for immune mediation, while the upstream mechanisms accounting for regulatory effects on energy metabolism are poorly understood. Once the function of quality‐control for protein processing is disrupted, ER acquires a stress state to and elicits the unfolded protein response to participate in a variety of pathophysiological process implicated in cellular apoptosis, oxidative stress, mitochondrial dysfunction, autophagy, etc.^[^ ^29^, ^30^, ^31^ ^]^ Herein, we discovered that KD, extracted from the herb medicine *Anoectochilus roxburghii*, remarkably restrained inflammation development and glycolytic activities in TMAO‐induced macrophages. The further mechanistic analysis demonstrated that KD bound to IRE1α to suppress the activation of IRE1α/XBP1 axis and subsequently repressed the expression of HIF‐1α governing the transcription of HK2, PKM2 and LDHA, thereby abrogating glycolysis‐induced inflammation progression. It should be noted that KD treatment also facilitated the process of TCA cycle and enhanced mitophagy activity, both of which were reversed following the administration of IRE1α agonist. These findings, along with fact that mitophagy not only served as the potential target of ER stress but also provided beneficial roles in OXPHOS development,^[^ ^32^, ^50^ ^]^ enlightened that KD‐triggered IRE1α/XBP1 pathway obstruction might increase the activity of mitophagy to accelerate the generation of TCA cycle metabolites and sequentially activate anti‐inflammatory cascades, thus potentiating the effects on driving macrophage repolarization toward M2 phenotype.

An ever‐growing wealth of evidence illustrates that the angiogenic function in the wound area display vital roles in determining the tissue repair speed.^[^ ^45^, ^46^ ^]^ Our results showed that KD obviously heightened proliferation and migration of HUVECs via reversing the pro‐inflammatory condition created by TMAO‐evoked macrophages and elevated vascular density in the diabetic wound region, which might be attributed to the increase of VEGFA and IGF‐1 expression and Notch pathway activation. Given that vascular endothelial cells developed unfavorable behaviors like early apoptosis, poor proliferation and slow migration in response to inflammation insult and M2 macrophages are able to release growth factors to propel wound repair, there existed dual functions elicited by KD in angiogenesis promotion.

Evidence from laboratory statistics has established that hydrogel biomaterials acting as drug delivery carriers are broadly used in the management of damaged tissue repair, by virtue of the high biocompatibility, low immunogenicity and desirable plasticity.^[^ ^5^, ^46^ ^]^ As expected, we fabricated a HAQA‐MN patch with excellent mechanical stress, controlled release and favorable safety. Since the burst release and rapid clearance properties confined the application of drugs in vivo, we introduced KD into the MN and found that this delivery vehicle prominently increased the bioavailability of KD, as reflected by the heightened effectiveness in the anti‐inflammatory capacity. Intriguingly, the needle‐like structure achieved the purpose of uniform spatial distribution of the cargo. This trait, together with the targeting ability conferred by cellular membrane coating method, further elevated the therapeutic efficiency of KD for diabetic wound repair.^[^ ^51^ ^]^

## 4 Conclusion

While the results are promising, further studies are needed to fully explore the clinical potential of the M‐KD‐loaded MN system. Future researches should be focused on optimizing the MN design for different wound types and sizes, assessing long‐term safety and efficacy, and conducting clinical trials to validate the system's performance in human patients. Additionally, exploring the potential of combining this MN system with other therapeutic modalities could further enhance its effectiveness in wound management. In conclusion, the HAQA‐based MN system loading KD encapsulated within a macrophage membrane represents a novel and promising approach for diabetic wound treatment. By addressing key challenges such as inflammation, infection, and oxidative stress, this multifunctional MN platform offers a comprehensive strategy for improving wound healing and managing chronic wounds.

## 5 Experimental Section

### Ethics Statement

The animal study was performed in line with the National Institutes of Health Guide for the Care and Use of Laboratory Animals and approved by the Institutional Animal Care and Use Committee of the Tongji Medical College, Huazhong University of Science and Technology (IACUC Number: 4004). The collection of wound samples from patients was approved by the Institutional Review Board at Union Hospital, Tongji Medical College, Huazhong University of Science and Technology (UHCT‐IEC‐SOP‐016‐03‐01).

### Statistical Analysis

The data in this study were presented as the mean ± standard deviation (SD) and were analyzed using Graphpad Prism software (version 9.5.1, CA, USA). Data showed a continuous normal distribution. The sample size (n) of each experiment was described in the corresponding figure legends. Differences comparison between two groups were performed using a two‐tail unpaired Student's t‐test. Statistical analyses of multiple groups were accomplished using one‐way ANOVA followed by Tukey's post hoc test. *p < 0.05, **p < 0.01 and ***p < 0.001 were considered to indicate statistical significance.

## Conflict of Interest

The authors declare no conflict of interest.

## Supporting information



Supporting Information

Supplemental Video 1

Supplemental Video 2

## Data Availability

The data that support the findings of this study are available from the corresponding author upon reasonable request.
