# Supplementary material for: Kinsenoside‐Loaded Microneedle Accelerates Diabetic Wound Healing by Reprogramming Macrophage Metabolism via Inhibiting IRE1α/XBP1 Signaling Axis
Source: Adv Sci (Weinh). 2025 Apr 25;12(26):2502293. doi: 10.1002/advs.202502293 (PMC12245111; doi:10.1002/advs.202502293)
Supplement: Supplementary file 1 — Supporting Information [file ADVS-12-2502293-s001.docx]

Materials and methods

*Reagents*: KD (purity > 99.8%) was obtained from MedChemExpress (Monmouth Junction, NJ, USA) and was dissolved in phosphate buffer saline (PBS, Gibco, NY, USA). Flow cytometry anti-CD11b (FITC), anti-F4/80 (APC), anti-CD86 (PE) and anti-CD206 (PerCP) were came from Biolegend, Inc. (San Diego, CA, USA). The IRE1α inhibitor 4μ8C and agonist IXA4 were purchased from MedChemExpress (Monmouth Junction, NJ, USA). DMAO/PI and Calcein/PI viability assay kit and EdU cell proliferation kit were acquired from Beyotime (Shanghai, China). Matrigel Matrix was purchased from Corning, Inc. (NY, USA). Primary antibodies for iNOS, CD163, IL-6, TNF-α, MCP-1, IL-10, CD31, Dll4, HIF-1α, IRE1α, p-IRE1α, HK2, PKM2, LDHA, IDH1 and CPT-1A were obtained from ABclonal (Wuhan, China). Primary antibodies for Arg-1, IL-4, TGF-β, p53, VEGFA, IGF-1, GAPDH and VDAC1 were purchased from Proteintech (Wuhan, China). Primary antibodies for p65, p-p65, STAT1, p-STAT1, STAT6, p-STAT6, Notch1, HES1, LC3, PINK1, Parkin, p62 and XBP1s were obtained from Cell Signaling Technology (Danvers, MA, USA). Primary antibodies for PCNA, CDK4, CDK6, cyclin D1, CD11b, integrin β1, ICAM1, VCAM1 and CCR2 were acquired from Affinity Biosciences (Changzhou, China). DHE fluorescence probe kit was obtained from Beyotime (Shanghai, China). Reverse transcription kit and SYBR-Green PCR Master Mix were purchased from Vazyme Biotech Co., Ltd. (Nanjing, China).

*Cell Culture*: Bone marrow derived macrophages (BMDMs) were acquired from the mice. Briefly, the femur and tibia were obtained from healthy male Balb/c mice aged 6 weeks. Via rinsing the marrow cavity, bone marrow cells were collected and resuspended in Dulbecco's Modified Eagle Medium/F12 (Gibco, NY, USA) supplemented 10% heat-inactivated fetal bovine serum (FBS, Cyagen Biosciences Inc., USA) and 40 ng/mL murine recombinant M-CSF (PeproTech, NJ, USA). After culture for 7 days in a 37 °C incubator containing 5% CO2, cells were differentiated into BMDMs which then were used for the following experiments.

Human umbilical vein endothelial cells (HUVECs) and L929 cells were obtained from the Cell Bank of the Chinese Academy of Science, Shanghai, China and maintained in RPMI-1640 (Gibco, NY, USA) containing 10% FBS. The culture medium was renewed every 2 days, cells at logarithmic growth stage were collected for in vitro experiments.

*Sample Collection*: The clinical wound tissues were obtained from diabetic patients and patients suffering foot trauma without other underlying diseases. All participants were Asian male adults aged 50-60 and the informed consent was obtained from them. All samples were collected from the Department of Orthopedics, Union Hospital, Tongji Medical College, Huazhong University of Science and Technology, Wuhan, China.

The murine wound tissues were acquired from diabetic mice and normal mice. Briefly, male Balb/c mice aged 6 to 8 weeks were obtained from Hubei Biont Biological Technology Co., Ltd (Wuhan, China). Animals were maintained under specific pathogen-free conditions, 12h light-dark cycle, diet and water were provided ad libitum. Mice were divided into the high fat diet group and normal chow diet group. Following 4 weeks of high-fat diet, the mice were intraperitoneally administrated with streptozotocin (STZ, 60 mg/kg/day) for 3 days to establish the diabetes model. After the concentration of the random blood glucose exceeded 16.7 mmol/L for two weeks, the mice were selected for the next experiment. Then, both of the diabetic mice and normal mice were then anesthetized by intraperitoneal injection of pentobarbital sodium (50mg/kg) and a full-thickness excisional skin wounds with a diameter of 10 mm was made on the dorsum. Subsequently, wound tissues of the two groups were gathered at post-operative day 7 for the next step.

*Detection of TMAO*: The obtained wound tissues were rinsed by ice-cold PBS to remove the residual blood. Then, on the ice, tissues were cut into pieces and mixed with PBS containing protease inhibitor at a mass volume ratio of 1:9. The mixture was sufficiently ground and subjected to the ultrasonication. After that, the homogenate was centrifuged at 5000 g for 10 min and the supernatant was collected for the quantification of TMAO using the ELISA kit according to the manufacturer’s protocols (ELK Biotechnology, Wuhan, China).

*Flow Cytometry*: Following the elimination of subcutaneous fat, 20 mg of skin wound tissues were immersed in RPMI-1640 and chopped into small pieces with the MACS dissociator (Miltenyi Biotech, CA, USA). Collagenase type IV and deoxyribonuclease I was added to digest the tissue at 37 ℃ for 1 h. The digestion was stopped by FBS and a 70 μm filter was used to obtain the single cell suspension, which was then centrifugated at 500 g for 5min. Cell pellet was washed with ice-cold PBS and delt with unlabeled anti-CD16/32 antibody to block Fcγ3 peptide. After twice of washing, cells were stained with CD11b, F4/80 and CD86 diluted in FACS buffer for 30 min at 4 °C. The suspension was centrifuged and cells were incubated with the fixation/permeabilization solution (BD Biosciences, NJ, USA) for 20 min at 4 °C. Following the rinse and incubation with CD206, cells were acquired on the LSRFortessa X-20 cytometer and data analyses were performed by FlowJo software.

BMDMs were distributed into individual wells of a 6-well plate and exposed to TMAO (50 μM) with/without incubation with varying concentration of KD (10, 20 μg/mL) for 24 h. Subsequently, cells were collected and flow cytometry was performed in accordance with the above staining steps.

*Plasmid Transfection and RNA Interference*: Plasmids encoding pcDNA3.1-FLAG-XBP1 and pcDNA3.1-FLAG-HIF-1α were procured from Genescript Biotechnology (Nanjing China). The constructed 1 μg plasmids per well were transiently transfected into BMDMs in 6-well plates with Lipofectamine 3000 kit (Invitrogen, CA, USA) according to the protocols. Knockdown of mRNA expression in BMDMs was carried out via cultivating cells in the 6-well plate and transfected with 30 pmol siRNA oligonucleotides via Lipofectamine RNAiMAX reagent (Invitrogen, CA, USA) in accordance with the manufacturer’s instructions. The sequences of siRNA targeting XBP1 and HIF-1α synthesized by GenePharma (Shanghai, China) were listed in Table S1 (Supporting Information).

*Lactate Generation, α-KG Production and Glucose Consumption Measurement*: The obtained cutaneous wound tissues were mixed with extraction solution, disaggregated on the ice and centrifuged at 12000 g for 10 min at 4 ℃. The supernatant was collected and applied for the determination of lactate and α-KG level according to the manufacturer’s recommended guidelines (Solarbio, Beijing, China).

For the analysis of intracellular lactate and α-KG production and glucose consumption, BMDMs were seeded in the 6-well plate and treated with different dosages of TMAO and KD for 24 h. Then lactate and α-KG colorimetric assay kit (Solarbio) and glucose uptake colorimetric assay kit were used (Biovision, SFO, USA) respectively as per each manufacturer’s recommendations.

*Seahorse Assay*: After BMDMs experienced indicated treatments, cells were seeded into the 96-well Seahorse XF cell culture microplates at a density of 1 × 10^5^ cells per well. Serial dosages of glucose (10 mM), oligomycin (2 μM) and 2-DG (50 mM) were added for the evaluation of ECAR and oligomycin (2 μM), FCCP (1 μM) and rotenone/antimycin A (0.5 μM) were used for the assessment of OCR with the XF96 Extracellular Flux Analyzer (Seahorse Biosciences) in accordance with the manufacturer’s protocols.

*RNA-Seq*: TMAO-stimulated BMDMs were treated with different dosages of KD (0, 20 μg/mL) for 24 h. Cellular total RNA was extracted using TRIzol Reagent (Invitrogen, CA, USA) and RNA integrity was evaluated by the RNA Nano 6000 Assay Kit of the Bioanalyzer 2100 system (Agilent Technologies, CA, USA). Then library quality was assessed on the Agilent Bioanalyzer 2100 system and RNA sequencing was performed on an Illumina Novaseq platform. Data analysis was carried out via the DESeq2 R package (1.20.0) and genes possessing an adjusted P-value < 0.05 and absolute foldchange > 2.0 witnessed by DESeq2 were considered as significantly differentially expressed. Gene set enrichment analysis was carried out using the local version of the GSEA tool (<http://www.broadinstitute.org/gsea/index.jsp>), NOM p-value < 0.05 and FDR q-value < 0.25 was considered statistically significant. The STRING database was applied for the protein-protein interaction analysis of differentially expressed genes.

*ChIP Assay*: The ChIP assay was performed using an EZ-ChIP kit (Millipore, USA) in accordance with the manufacturer’s protocols. BMDMs were fixed in 1% formaldehyde at room temperature for 10 min, followed by lysis and sonicated processing. The antibody against XBP1s and rabbit IgG was used and the precipitated DNA was subjected to quantitative PCR. The primes used in the ChIP assay were listed in Table S2.

*Dual-Luciferase Reporter Assay*: Cells were transfected with pGL3 reporter carrying the wild-type or mutant HIF-1α promoter and pRL-TK vectors (Promega, USA). After being cultured for 48 h, cells were harvested for measuring the luciferase activity using the dual-luciferase activity reporter assay system (Promega, USA) according to the manufacturer’s instructions.

*Pull-Down Assay*: Cellular lysates from BMDMs were incubated with biotin, biotin-KD or the combination of biotin-KD and KD at 4 ℃ overnight. Afterwards, streptavidin-conjugated beads were used for the pull-down procedure. Following 4 h of incubation, the beads were washed with PBS and subjected to be boiled in loading buffer. The supernatant was acquired and loaded on the SDS-PAGE for western blot analysis of IRE1α.

*Molecular Docking*: The chemical structure of KD was prepared from the PubChem database (https://pubchem.ncbi.nlm.nih.gov/). The crystal structure file of IRE1α was downloaded from the RCSB Protein Data Bank (PDB, <https://www.rcsb.org/>). The obtained structures were processed including de-watering, polar hydrogen atom addition and electron transfer with PyMOL software (version 2.3.2, NY, USA). Subsequently, docking analysis was carried out by AutoDock Vina 1.1.2 and docking model was visualized using PyMOL software.

*Cellular Thermal Shift Assay (CETSA)*: BMDMs were gathered and free-thawed three times with liquid nitrogen. The cell lysates were then incubated with KD (20 μg/mL) or DMSO at room temperature for 1 h. Afterwards, cell lysates were heated independently at designated temperatures (ranging from 46 to 61℃) for 5 min and then cooled to room temperature. The supernatant was obtained by centrifuging at 20000 g for 15 min and subjected to western blot analysis.

*Drug Affinity Responsive Target Stability (DARTS)*: BMDMs were scraped from the culture dish and mixed with the lysis buffer. The supernatant was acquired by centrifugation and incubated with different concentrations of KD (0, 5, 10, 20 μg/mL) for 1 h. Then the pronase (Sigma-Aldrich) at a ratio of 1:1000 was added for additional 30 min at room temperature and protease inhibitor was applied to terminate the reaction. Western blot was employed to evaluate the level change of IRE1α.

*Cell Proliferation Assay*: HUVECs were cultured in the lower chamber of a 24-well plate at a density of 1 × 10^5^ per well. BMDMs pretreated with or without TMAO (50 μM) and KD (20 μg/mL) were seeded in the upper chamber. After 24 h of incubation, EdU assay was performed to assess the proliferative ability of HUVECs.

*Cell Scratch Test*: HUVECs were cultured into the lower chamber of a 6-well plate and scratched with a sterile 1-mL pipette tip when the growth density reached approximately 90%. Floating cells were removed and HUVECs were incubated with primed BMDMs for 24 h. The remaining scratch area was photographed and measured using ImageJ software.

*Transwell Assay*: The 24-well incubation plates inserted with 8-μm-pore-sized filters was used to evaluate the migratory ability of HUVECs. BMDMs were subjected to indicated experimental processes prior to performing the assay. 1 × 10^4^ HUVECs were suspended in low-serum (5% FBS) medium and plated into the upper chamber. BMDMs suspended in complete medium (10% FBS) were seeded into the lower chamber. Following 24 h of incubation, HUVECs migrated on the bottom side of the filter were fixed and stained with 0.1% crystal violet, and then were captured under an optical microscope (Olympus, Japan).

*Tube Formation Assay*: For the tube formation detection, 250 μL of cold Matrigel was added to each well of a 24-well plate and maintained at 37 °C for 1 h. Subsequently, HUVECs (2 × 10^4^ per well) were plated on the Matrigel-coated plate and co-incubated with pretreated BMDMs in the upper chamber for 6 h. The tube length and total branch points were observed and determined by ImageJ software.

*Synthesis of M-KD*: For the isolation of macrophage membranes, BMDMs were suspended in a homogenization buffer containing 10 mM KCl, 2 mM MgCl_2_, 75 mM sucrose, 20 mM Tris-HCl (pH 7.5) and protease/phosphatase inhibitor. Cells were broken up via an ultrasonic disruptor for 10 min at 4 ℃, accompanied by centrifugation at 20000 g for 25 min at 4 ℃. The supernatant was discarded and cellular membranes were collected and stored at -80 ℃ for further usage. Then, the M-KD was developed via co-extruding KD and macrophage membranes through 800 nm and 400 nm polycarbonate filters approximately 15 times. The protein profiles of coated membranes were determined by the Coomassie brilliant blue staining complying with the protocols (Vazyme Biotech). Moreover, specific biomarkers associated with the recognition and function of the membrane was confirmed by western blot analysis.

For the detection of drug loading efficiency, different dosages of KD were dissolved in the methanol and then exposed to the ultra violet. The obtained absorbance value was used to form calibration curve (y = 0.02574 * x – 0.00084), which in turn calculated the drug loading efficiency of M-KD.

For the affinity ability measurement, Cy3 labelled M-KD was synthesized via co-incubating the membrane with Cy3 fluorescent dye in the ethanol. Then Cy3-M-KD was added into the culture medium of BMDMs, HUVECs and L929 cells respectively. Following 6 h of incubation, cells were harvested and subjected to the detection of fluorescence intensity.

*Synthesis of Hydrazide Group Grafted HA (HA-ADH)*: Briefly, MES buffer was formed by dissolving 1 g 2-(N-Morpholino) ethanesulfonic in 200 ml DI and adding sodium hydroxide to adjust the pH to 6.5. Next, add 1 g HA to 100 ml MES buffer and the mixture was stirred to mix well. Then 1.25 g EDC and 0.89 g HOBT were added to the solution for 1 h reaction to deactivate the carboxyl group in HA. 4.5 g ADH was added to the mixture and reacted at room temperature for 24 hours. Finally, the mixture was dialyzed with DI water (14 kDa cut-off dialysis membrane) for 4 days and freeze-dried to obtain HA-ADH.

*Synthesis of Aldehyde HA (HA-ALD)*: First, 2 g HA was dissolved in 200 ml DI water. A total of 1.2 g NaIO_4_ was then added and stirred for 24 h under dark conditions. Then 2 ml ethylene glycol was added to terminate the oxidation. The resulting mixture was dialyzed against DI water for 4 days to remove unreacted reagent (14 kDa cut-off dialysis membrane). Finally, the liquid was lyophilized for 3 days to obtain HA-ALD.

*Synthesis of Quaternary Ammonium and Aldehyde Grafted HA (HA-QA-ALD)*: 0.4 g Girard’s reagent T was added to 100 ml 1% (w/w) HA-ALD solution. The pH of the mixture was adjusted to 4.5 with 1M sodium hydroxide solution. The mixed solution was stirred and reacted for 24 hours at room temperature. In order to obtain dry HA-QA-ALD, the mixture was dialyzed with DI water (14 kDa cut-off dialysis membrane) for 4 days and finally freeze-dried.

*Preparation of HAQA Hydrogel*: The same volume of HA-ADH and HA-QA-ALD were added to the mold and mixed evenly to obtain HAQA hydrogel. We prepared hydrogels with final solid concentrations of 2%, 4% and 6% respectively for subsequent studies.

*Micro-morphologies Scan*: The micro-morphologies of freeze-dried hydrogel samples were observed via scanning electron microscopy (Quattro S, America). These samples were freeze-dried and quenched with liquid nitrogen, then quickly exposed to cross sections and sprayed with gold.

*Swelling Property*: To assess swelling, hydrogel samples (100μL per gel, n=4) were prepared and the initial weight of W_0_ was recorded. Then the samples were immersed in 2mL PBS at 37 ° C for 10 hours. At the end of the immersion, water was removed from the sample surface, and the weight was recorded as W_t_. The swelling ratio (%) was calculated according to the formula:

Swelling Ratio=$W_{t}/W_{0}\times100\%$_._

*Rheological Test*: The dynamic property of HAQA hydrogel were tested by rheological test by using rotary rheometer (Anton Paar, MCR302). We used a plate model with a plate diameter of 25 mm and a plate spacing of 1 mm under 25℃. The time sweep was tested with the fixed frequency (1 Hz) and strain (1%). The strain sweep was tested with the fixed frequency (1 Hz) and the changed strain (1%-500%). For the low-high strain cyclic test, the frequency was fixed at 1 Hz, and the low and high strain was fixed at 1% and 300%, respectively.

*Synthesis of M-KD@HAQA-MN*: The M-KD was mixed with HA-ADH solution (4%) and HA-QA-ALD solution (4%) separately and stirred for 30 min to fully dispersed in the suspension. Then both of HA-ADH and HA-QA-ALD were added to the polydimethylsiloxane (PDMS) MN mold (Henan Micro-Nano BenTeng Biotechnology Co., Ltd., Henan, China), followed by gentle mixture, vacuum pumping and elimination of air bubble. After 5 min of cross-linking and curing, the MN patch was demolded and applied for the next experiment.

For visualizing the distribution of M-KD in the MN delivery vehicle and the skin tissue following penetration, Cy3-lablled M-KD and FITC-loaded HA-ADH and HA-QA-ALD solution were used. The MN system imaging was performed with the confocal laser scanning microscopy (Nikon, Japan). Additionally, the appearance of the M-KD@HAQA-MN was acquired using a stereomicroscope (Olympus, Japan) and scanning electron microscope (ZEISS, Germany). The murine and porcine dorsal skin tissues pierced by the MN array to ascertain the penetration property. The uniaxial compression test was carried out with a displacement-force test station (Hengyi, China) to assess the fracture force of the MN patch. Subsequently, the standard curve of KD absorbance was plotted and the quantification of KD release from the MN was performed using a microplate reader (Thermo Fisher, USA).

*Antimicrobial Activity Test*: *Escherichia coli* (E. coli, Gram-negative strains, ATCC 25922) and *Staphylococcus aureus* (S. aureus, Gram-positive strains ATCC 25923) were selected to evaluate the antibacterial property of M-KD@HAQA-MN. The bacterial suspension (10^7^ CFU/mL) was incubated and shaken with HAQA-MN or M-KD@HAQA-MN at 37 ℃ for 12 h and administration of PBS was set as a control group. Then, the treated bacterial solution was diluted with the same ratio and cultured on Luria-Bertani solid medium for further 12 h. The remaining colonies were photographed and counted to assess the survival ratio. In addition, following different incubation approaches, the bacterial solution was subjected to live/dead detection using the DMAO/PI staining kit according to the manufacturer’s protocols.

*ROS Level Detection*: The wound tissues were embedded and sectioned and then subjected to DHE staining following the manufacturer’s instructions. For the evaluation of intracellular ROS scavenging ability of the MN system, HUVECs and L929 cells were cocultured with or without the MN patch under the stimulation of H2O2 (100 μM) for 24 h, accompanied by the application of DCFH-DA reagent and analysis of the fluorescence intensity.

*Cell Viability*: HUVECs and L929 cells were seeded into the 96-well plates and incubated with PBS, HAQA-MN or M-KD@HAQA-MN for 24 h at 37 ℃. Subsequently, the Calcein/PI staining kit was used to examine the viability of cells. To determine whether the MN patch alleviated cellular oxidative injury, H2O2 (200 μM) added to the culture medium. After 24 h, Calcein/PI staining was performed to detect cellular survival and death.

*JC-1 Staining*: After BMDMs were treated with different interventions for 24 h, cells were stained with JC-1 kit (DOJINDO, Japan) to determine the mitochondrial membrane potential with the confocal laser scanning microscopy.

*Animal Study*: Male Balb/c mice aged 6 to 8 weeks were fed with high-fat diet and intraperitoneally injected with STZ to establish the diabetes model as above described. Then, a full-thickness skin defect model (diameter ≈ 10 mm) was created on the back of all animals. The wounds were randomly received PBS, KD (20 mg/kg), KD@HAQA-MN and M-KD@HAQA-MN separately and photographed at days 0, 3, 7, 10 post-operation. At day 14, mice were sacrificed and the skin tissues and internal organs were harvest for further analysis. The area of wound closure was assessed by ImageJ software.

For investigating the antibacterial effects of the MN patch on the healing process of the infected diabetic wounds, 50 μL of S. aureus suspension (1.0 × 10^6^ CFU/mL) was inoculated into the created wounds initially. After 24 h, the mice were randomly received PBS, KD (20 mg/kg), KD@HAQA-MN and M-KD@HAQA-MN separately. At day 14, mice were sacrificed and the area of wound closure was assessed by ImageJ software.

*Histopathological Analysis*: The harvested organ and wound tissues were fixed, dehydrated and embedded in paraffin. Samples were cut into 6 μm thick sections, accompanied by hematoxylin and eosin (H&E) and Masson’s trichrome (MASSON) staining.

*Biocompatible test*: The blood samples of mice were collected at day 14, which were then centrifuged and the upper serum was obtained. The values of Hb, WBC and PLT were determined by the blood routine analyzer (Mindray, China). The levels of AST, ALT and Cr were detected using kits from Nanjing Jiancheng Bioengineering Institute according to the manufacturer’s protocols. Then, the intervention agents in each group were added to the plasma of mice and subjected to the hemolytic test and ddH_2_O was set as the positive control.

*Blood Perfusion Detection*: At day 10 after the operation, the laser speckle contrast imaging system (PERIMED Ltd, Sweden) was used to measure the local blood flow in the wound region. A near-infrared laser at 785 nm was applied to detect blood perfusion, which was termed as perfusion unit. The mean perfusion units (MPU) ratio was calculated by comparing the MPU of the wound area with the MPU of the area surrounding the wound.

*Immunofluorescence Staining*: In brief, the fixed and permeabilized cells and epitope retrieval tissue sections were blocked with goat serum and then incubated overnight with primary antibodies at 4 °C. The samples were washed with PBS three times and stained with FITC or Cy3-conjugated secondary antibodies. The nuclei were incubated with DAPI and the images were captured by a confocal laser scanning microscopy.

*qRT-PCR*: Total RNA was extracted from cells and wound tissues using TRIzol reagent. The HiScript III RT Super-Mix was employed to reverse-transcribe the RNA into cDNA following the manufacturer’s instructions. Real-time PCR was executed on a Light-Cycler 480 II using 2x SYBR Green qPCR Mix. The 2^−ΔΔCt^ method was used to quantify relative mRNA expression and GAPDH was used to normalize mRNA levels. Primer sequences were listed in Table S2.

*Western blot*: The total proteins and mitochondrial proteins from cells and tissues were extracted with kits from Beyotime according to the protocols. After quantification, protein samples were separated by 10% SDS-PAGE and then transferred onto polyvinylidene difluoride membranes. Then membranes were blocked with 5% BSA at room temperature for 1 h, followed by incubation with primary antibodies at 4 °C overnight. Subsequently, membranes were washed three times with tris-buffered saline-tween 20 and incubated with corresponding secondary antibodies for 1 h at room temperature. The protein bands were visualized by the electrochemiluminescence kit (Vazyme Biotech, Nanjing, China) and the gray values of the bands were quantified and analyzed using ImageJ software.

Table S1 The list of siRNA sequences in this study

|  | Forward strand (5'-3') | Reverse strand (5'-3') |
| --- | --- | --- |
| si-XBP1 | GGAAGAAGAGAACCACAAACU | UUUGUGGUUCUCUUCUUCCAA |
| si-HIF-1α | CCAUGAUAUGUUUACUAAAGG | UUUAGUAAACAUAUCAUGGUG |

Table S2 The primer sequences in this study

|  | Forward (5'-3') | Reverse (5'-3') |
| --- | --- | --- |
| Primer sequence for ChIP | |  |
| HIF-1α | TTCCCCCGTCCACCCATTTC | GACTCTTTGCTTCGCCGAGA |
| Primer sequence for qRT-PCR | |  |
| Mice | | |
| IL-6 | CCCCAATTTCCAATGCTCTCC | CGCACTAGGTTTGCCGAGTA |
| MCP-1 | CCCCAAGAAGGAATGGGTCC | GCATCACAGTCCGAGTCACA |
| TNF-α | CCCACGTCGTAGCAAACCAC | GCAGCCTTGTCCCTTGAAGA |
| IL-4 | CCCCCAGCTAGTTGTCATCC | AGGACGTTTGGCACATCCAT |
| TGF-β | ACTGGAGTTGTACGGCAGTG | GGGGCTGATCCCGTTGATTT |
| IL-10 | GCTCCAAGACCAAGGTGTCT | AGGACACCATAGCAAAGGGC |
| HK2 | CAGGCTACCCGGAGTTGTTC | TGGTTTTGGTTGAGCTCCGT |
| PKM2 | TATCGCAGCAGGAACCGAAG | TCAGCCGAGCCACATTCATT |
| LDHA | TACAATCTACCGAGCGGAGG | AGCTCACTGCTGTGTTCCAA |
| XBP1 | GCAGCAAGTGGTGGATTTGG | ACATAGTCTGAGTGCTGCGG |
| HIF-1α | GCGGCGAGAACGAGAAGAAA | AATATGGCCCGTGCAGTGAA |
| VEGFA | GCAGCGACAAGGCAGACTAT | AAGAGCCCAGAAGTTGGACG |
| IGF-1 | CGAATGTTCCCCCAGCTGTTT | GTTTGTCGATAGGGACGGGG |
| NQO1 | AGCCAATCAGCGTTCGGTAT | GCCTCCTTCATGGCGTAGTT |
| CAT | TTTTGCCTACCCGGACACTC | GGGGTAATAGTTGGGGGCAC |
| Human | | |
| VEGFA | CCAAGATCCGCAGACGTGTA | TGACGATGATGGCGTGGTG |
| IGF-1 | TCCCACGGAGCAGAAAATGC | CAACAGCAATCTACCCACGC |
| NQO1 | AGCGAGTGTTCATAGGAGAGT | GCAGAGAGTACATGGAGCCAC |
| CAT | ACTTCTGGAGCCTACGTCCT | AAAGTCTCGCCGCATCTTCA |


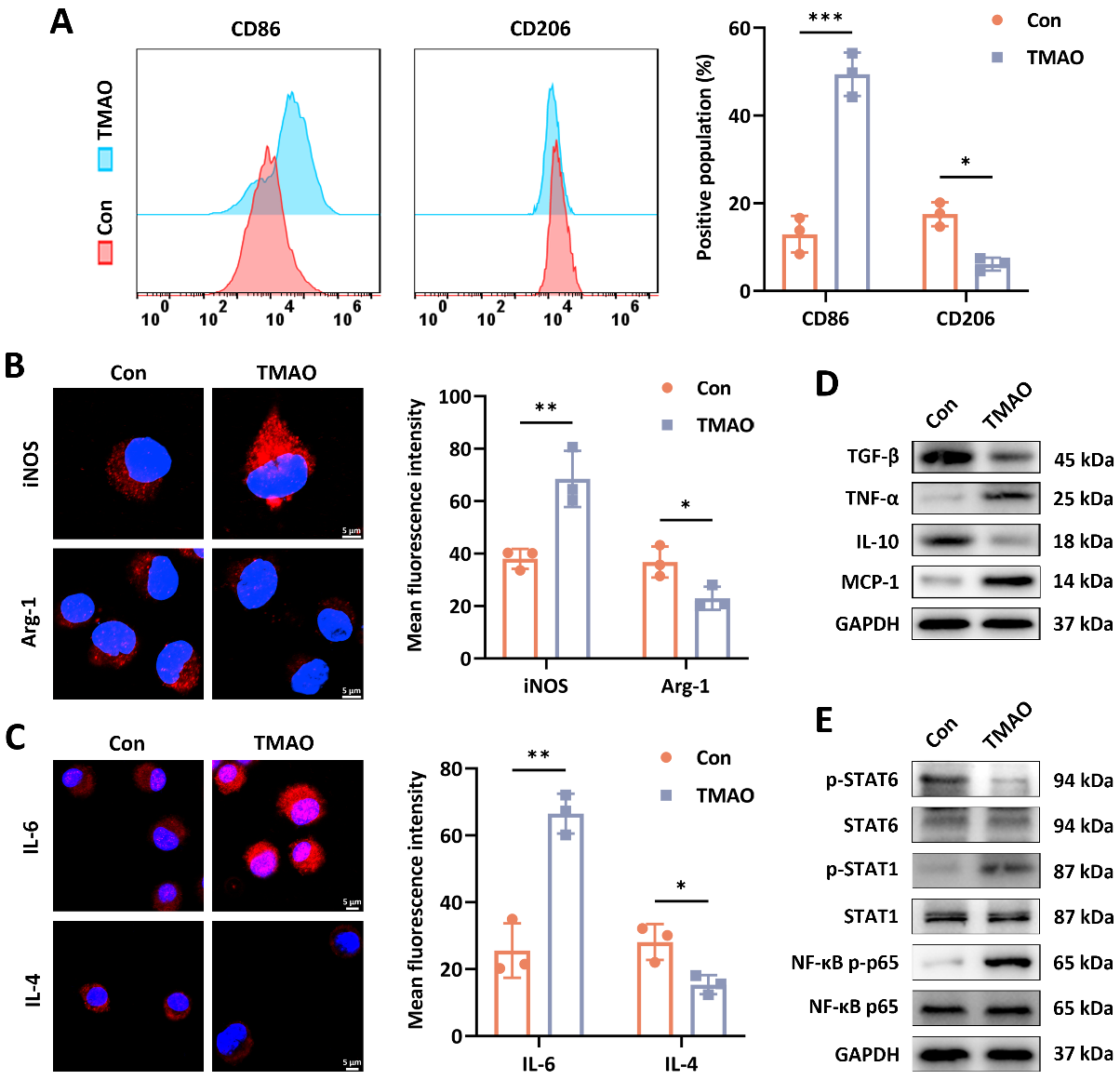


Figure S1. Effects of TMAO on the inflammation development of macrophages. A) Flow cytometry revealed the percentage of CD86+ and CD206+ populations among BMDMs (n = 3). B) levels of iNOS and Arg-1 and C) contents of IL-6 and IL-4 were assessed by immunofluorescence analysis (n = 3). D) The expression of TGF-β, TNF-α, IL-10 and MCP-1 and E) activity of signaling factor NF-κB, STAT6 and STAT1 was evaluated using western blot (n = 3). Data were presented as mean ± SD. Statistical significance was determined using Student’s t test. *p < 0.05, **p < 0.01, ***p < 0.001.


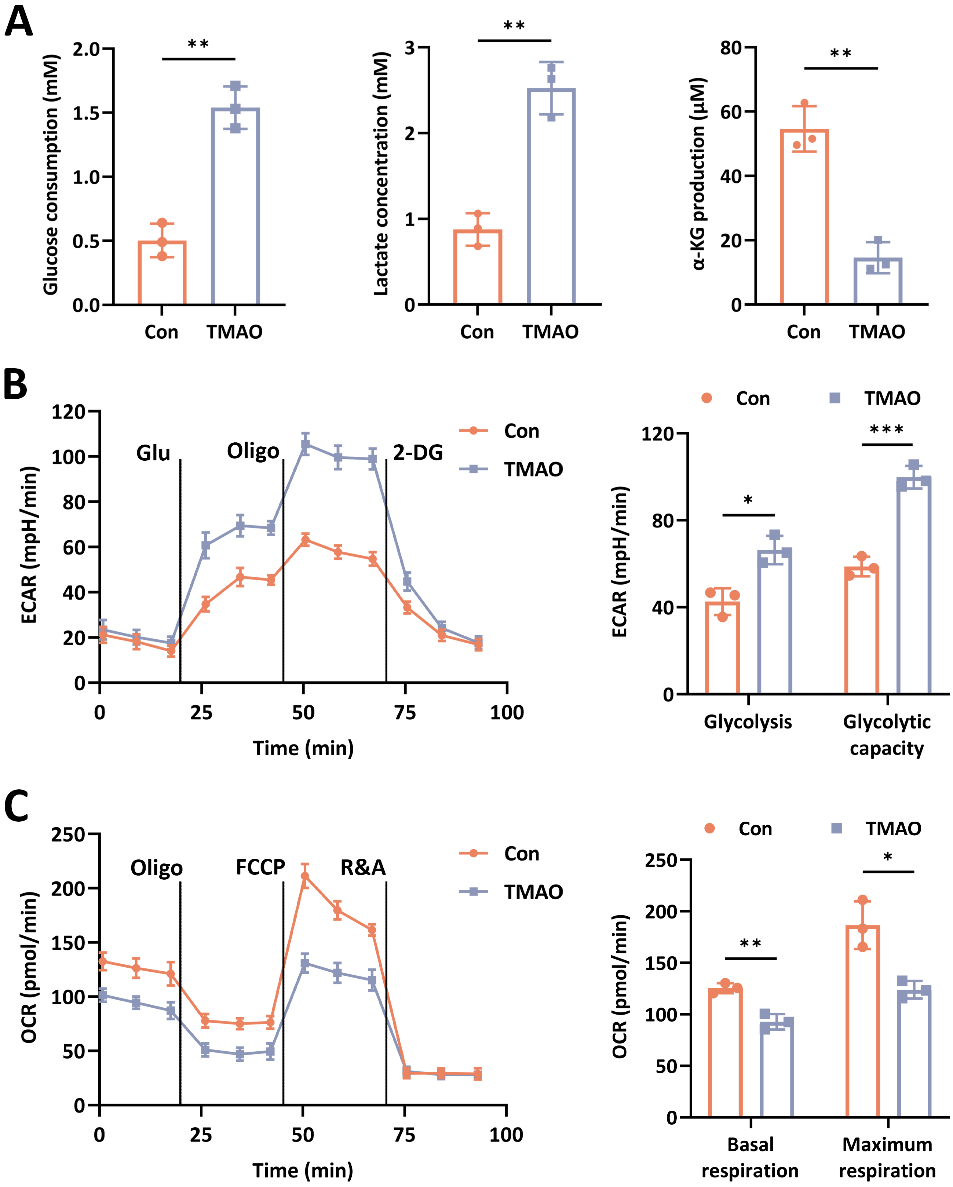


**Figure S2.** The effect of TMAO on the glycolytic activities of macrophages. A) Glucose uptake and lactate and α-KG production of BMDMs with or without TMAO intervention (n = 3). B) The value of ECAR and B) OCR of BMDMs in the two groups was measured by Seahorse assay (n = 3). Data were presented as mean ± SD. Statistical significance was determined using Student’s t test. *p < 0.05, **p < 0.01, ***p < 0.001.


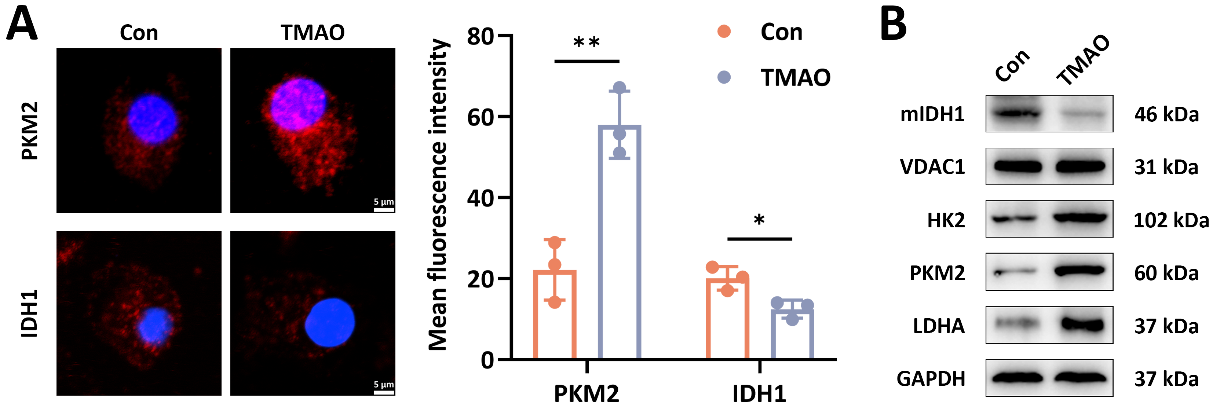


**Figure S3.** Level changes of metabolic enzymes in macrophages following TMAO administration. A) Immunofluorescence staining was used to detect the expression of PKM2 and IDH1 in BMDMs (n = 3). B) Protein contents of LDHA, PKM2, HK2 and mitochondrial IDH1 was measured using western blot (n = 3). Data were presented as mean ± SD. Statistical significance was determined using Student’s t test. *p < 0.05, **p < 0.01.


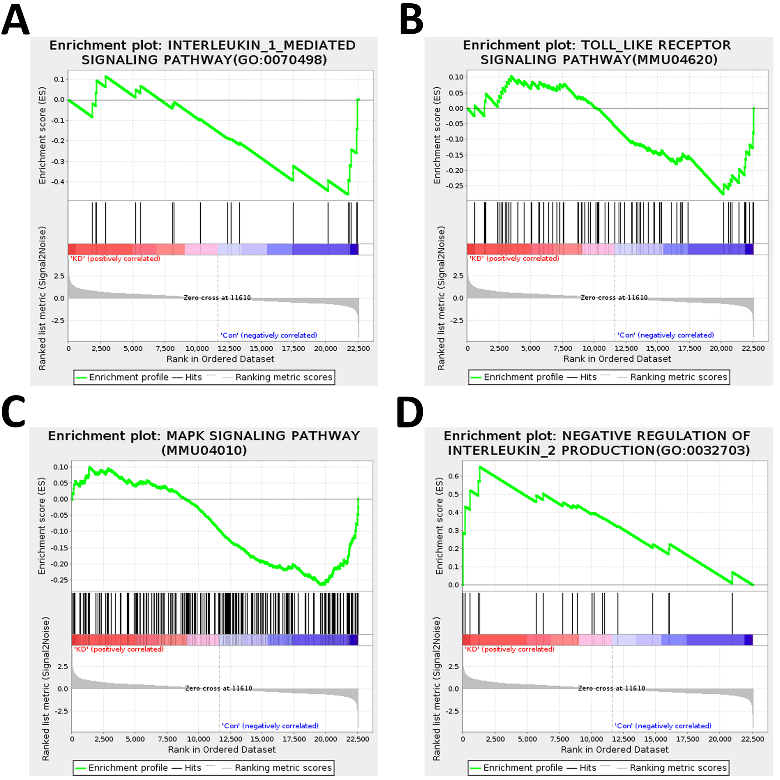


**Figure S4.** GSEA enrichment showed that KD treatment significantly inhibited the signaling pathways including IL-1(A), TLR (B), MAPK (C) and IL-2 (D).


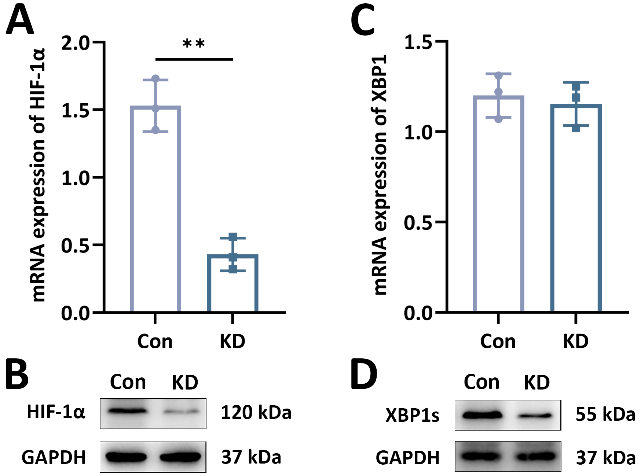


**Figure S5.** The regulatory roles of KD in HIF-1α and XBP1 expression of BMDMs stimulated with TMAO. A) mRNA level and B) protein content of HIF-1α in macrophages of the two group (n = 3). C) qRT-PCR and D) western blot was used to examine the expression of XBP1 in BMDMs (n = 3). Data were presented as mean ± SD. Statistical significance was determined using Student’s t test. **p < 0.01.

**
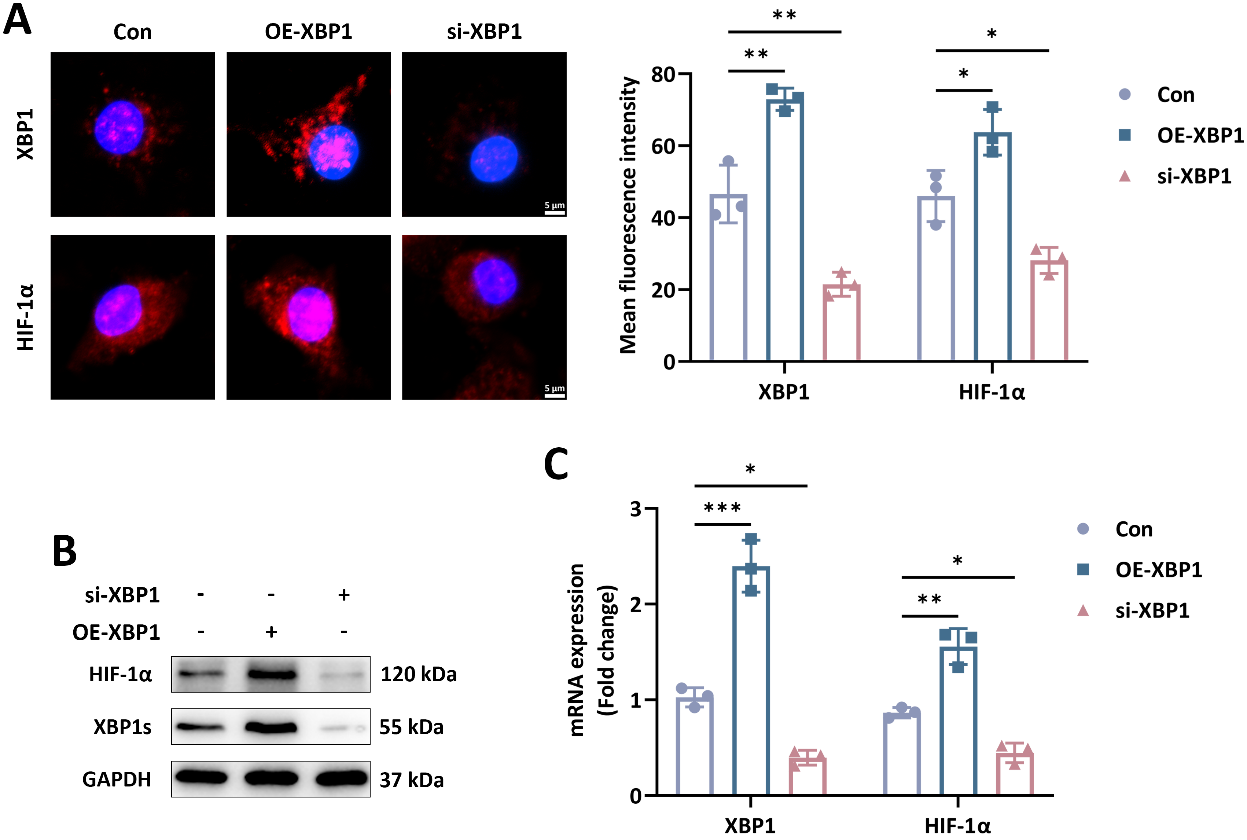
Figure S6.** Effects of XBP1 expression alteration on the level of HIF-1α macrophages. A) Immunofluorescence staining showed the expression of XBP1 and HIF-1α in BMDMs with or without the treatment of XBP1-overexpressing plasmid (OE-XBP1) and siRNA-XBP1 (si-XBP1) (n = 3). B) The protein levels and C) mRNA contents of XBP1 and HIF-1α in BDMDs with indicated treatments (n = 3). Data were presented as mean ± SD. Statistical analysis was performed using one-way ANOVA followed by Tukey’s post hoc test. *p < 0.05, **p < 0.01, ***p < 0.001.


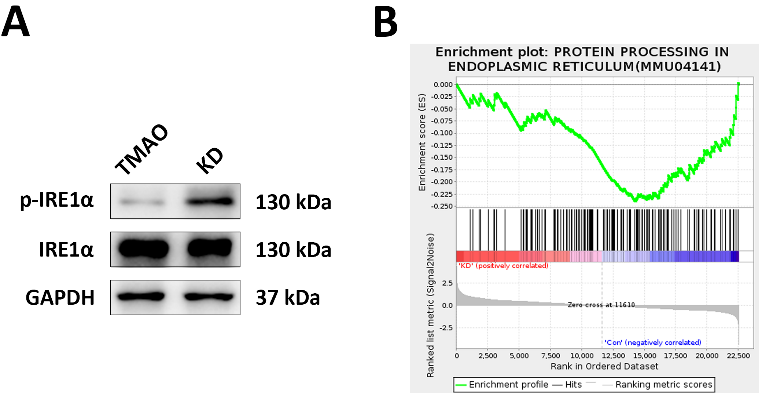


**Figure S7.** The regulator roles produced by KD in ER stress development of macrophages exposed to TMAO. A) Western blot was employed to analyze the activity of the ER stress initiator IRE1α in TMAO-induced BMDMs following KD intervention (n = 3). B) The activity of signal pathway involving protein processing in ER was determined by GSEA.


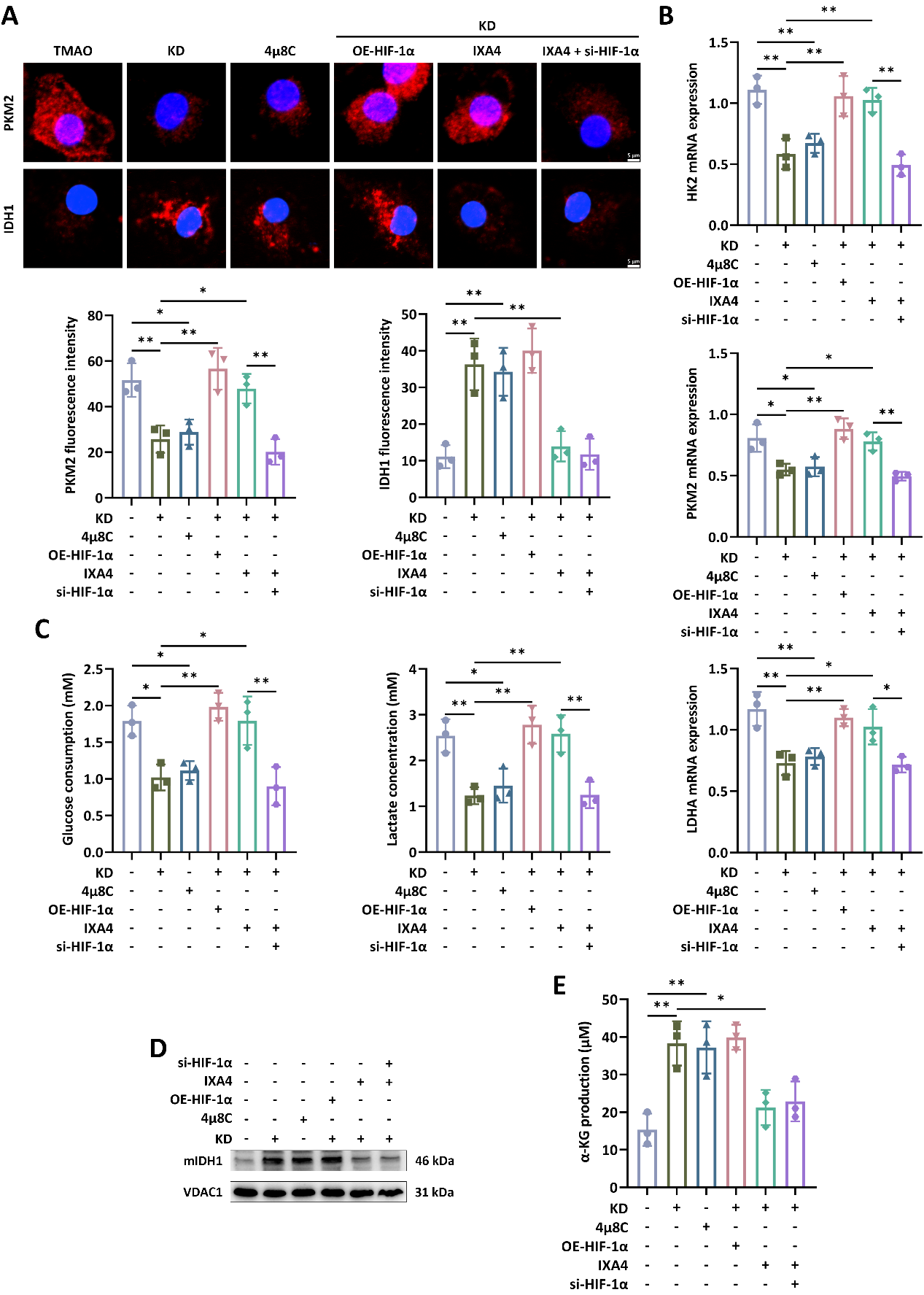
**Figure S8.** KD regulated XBP1/HIF-1α axis activity to affect the expression of glycolytic enzymes. A) Immunofluorescence staining revealed the expression changes of PKM2 and IDH1 in BMDMs following the activity regulation of XBP1/HIF-1α pathway (n = 3). B) The mRNA expression of HK2, PKM2 and LDHA was detected by qRT-PCR (n = 3). C) Alterations of glucose consumption and lactate generation of BMDMs with indicated treatments (n = 3). D) Western blot was used to detect the expression of mitochondrial IDH1 (n = 3). E) The α-KG production of BMDMs exposed to varying treatments (n = 3). Data were presented as mean ± SD. Statistical analysis was performed using one-way ANOVA followed by Tukey’s post hoc test. *p < 0.05, **p < 0.01.


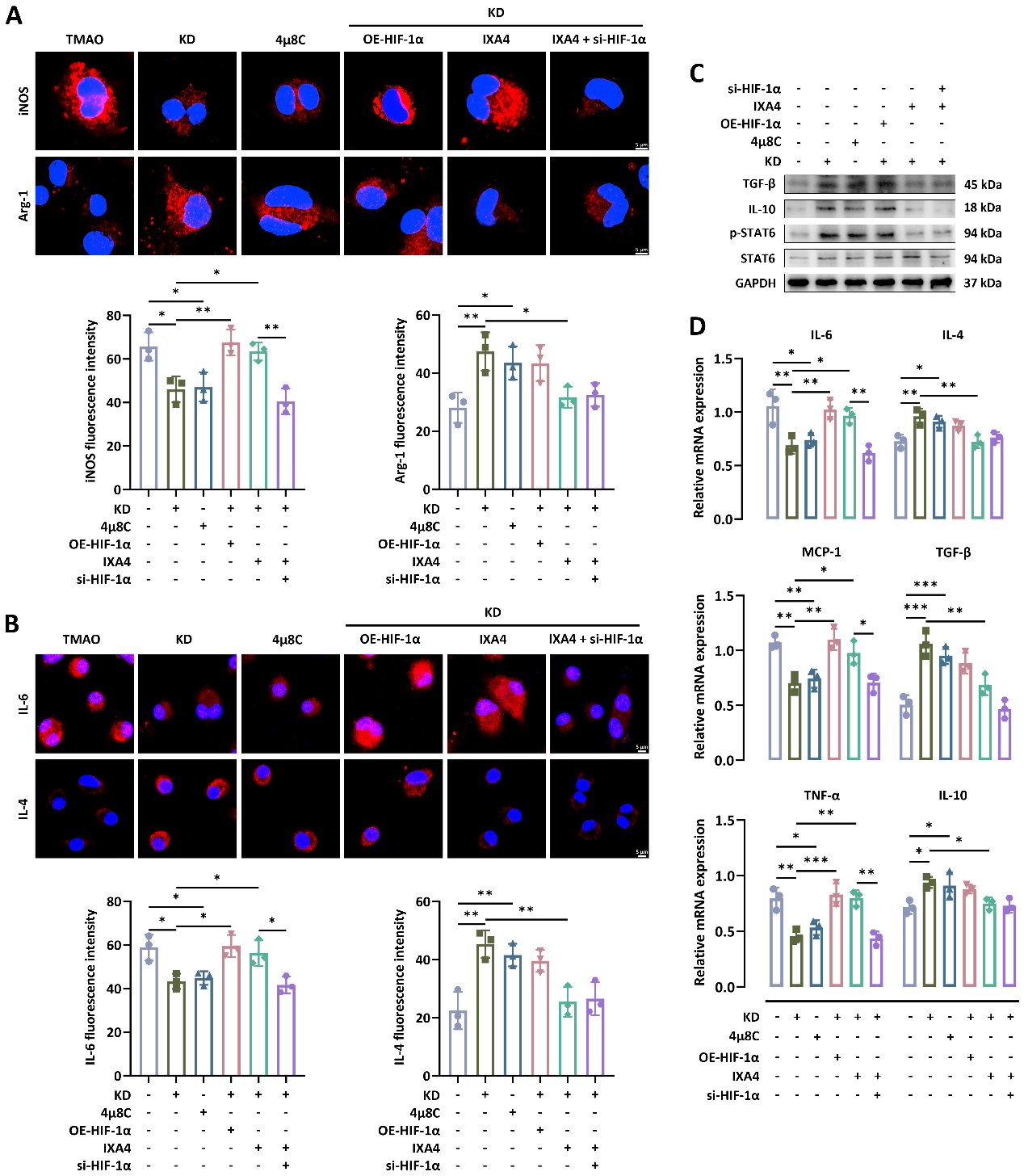


**Figure S9.** The effects of activity alteration of XBP1/HIF-1α pathway on the inflammation development of macrophages. A) expression of iNOS and Arg-1 and B) level of IL-6 and IL-4 was measured via immunofluorescence staining (n =3). C) Protein levels and D) mRNA contents of inflammation-related cytokines were detected (n =3). Data were presented as mean ± SD. Statistical analysis was performed using one-way ANOVA followed by Tukey’s post hoc test. *p < 0.05, **p < 0.01, ***p < 0.001.


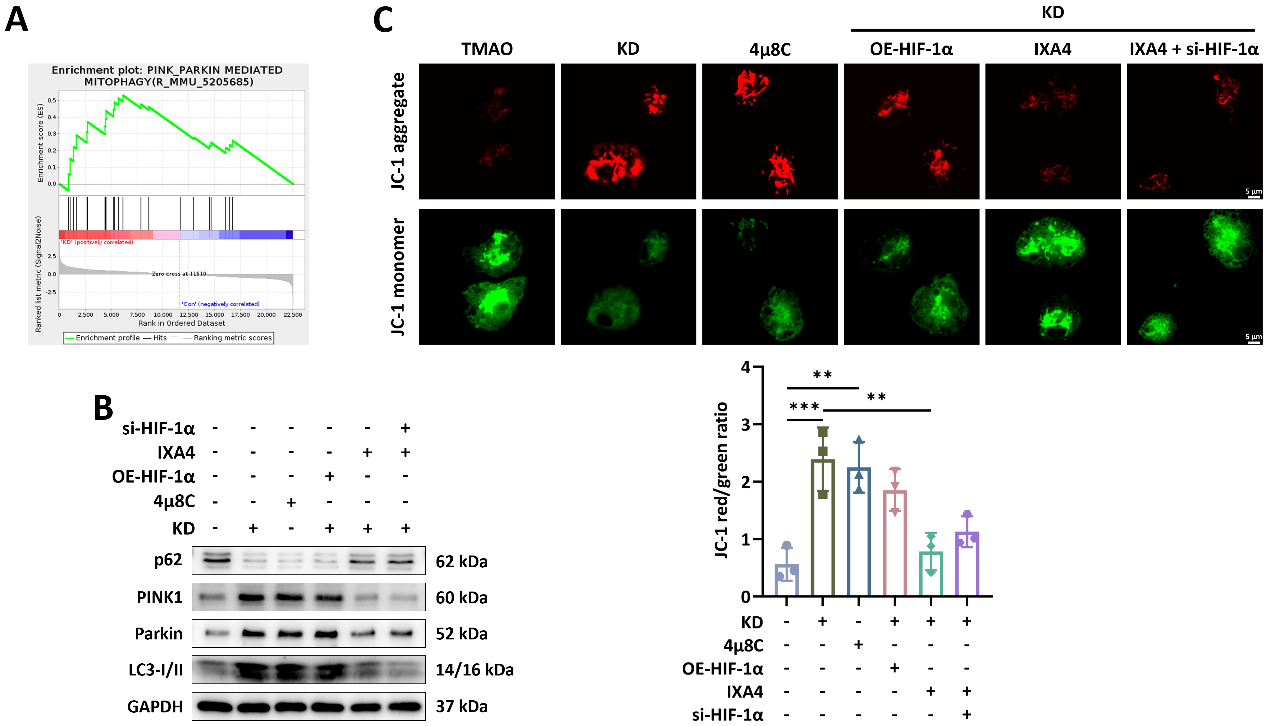


**Figure S10.** KD treatment suppressed ER stress to facilitate mitophagy development in TMAO-induced macrophages. A) The expression of gene sets associated with PINK/Parkin-mediated mitophagy was detected using GSEA. B) Levels of PINK1, Parkin, p62 and LC3I/II in BMDMs following indicated treatments (n = 3). C) The mitochondrial membrane potential in BMDMs was determined using JC-1 staining (n =3). Data were presented as mean ± SD. Statistical analysis was performed using one-way ANOVA followed by Tukey’s post hoc test. **p < 0.01, ***p < 0.001.





**Figure S11.** The Fourier transform infrared (FT-IR) spectrum of HA, HA-ADH, and HA-QA-ALD.


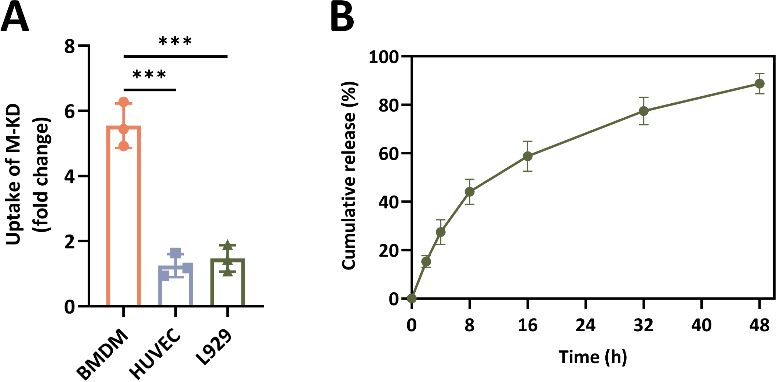


**Figure S12.** A) The relative level of M-KD engulfed by the cells (n = 3). B) Release curve of KD from the MN system in vivo (n = 3). Data were presented as mean ± SD. Statistical analysis was performed using one-way ANOVA followed by Tukey’s post hoc test. ***p < 0.001.


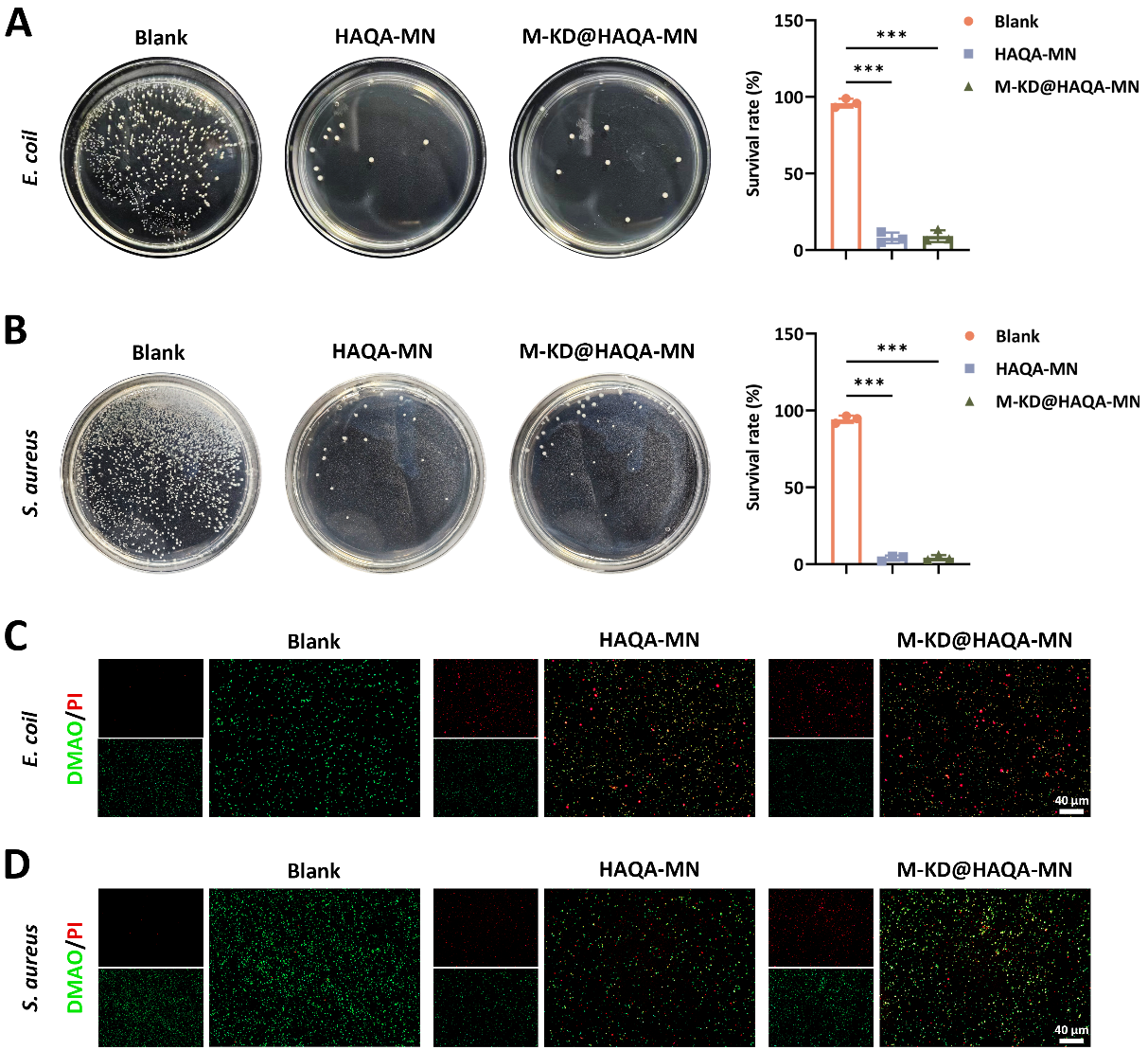


**Figure S13.** The antibacterial properties of the MN system against *E. coil* and *S. aureus* with the plate counting method (A,B) and live/dead assay (C,D) (n = 3). Data were presented as mean ± SD. Statistical analysis was performed using one-way ANOVA followed by Tukey’s post hoc test. ***p < 0.001.


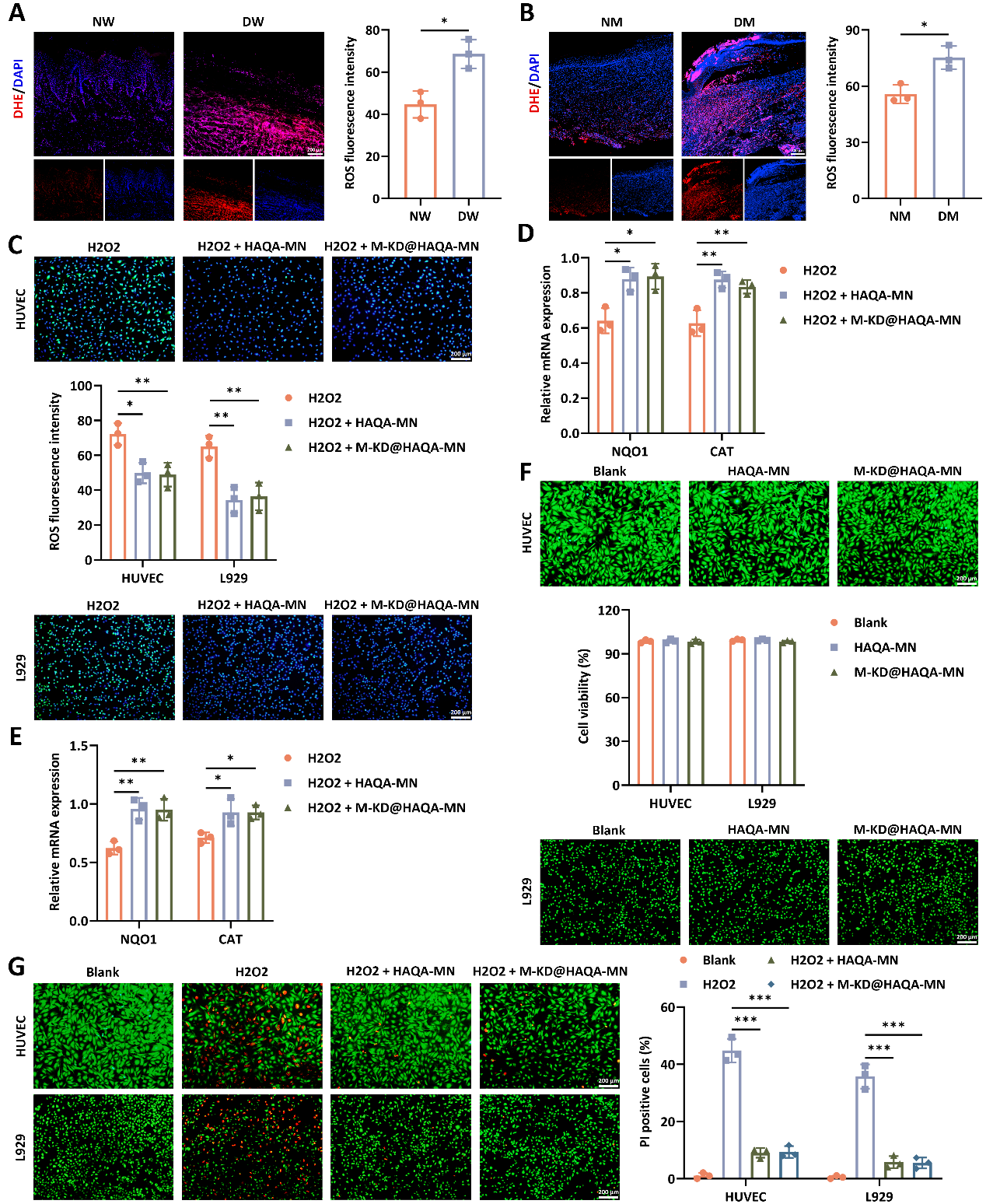


**Figure S14.** The ROS-scavenging abilities of M-KD@HAQA-MN. DHE staining was applied to evaluate the level of ROS in the normal and diabetic wound tissues from patients (A) and mice (B) (n = 3). C) Effects of the MN patch on ROS elimination in H2O2-insulted HUVECs and L929 cells were detected via DCFH-DA kits (n = 3). qRT-PCR was used to detect the mRNA level of NQO1 and CAT in HUVECs (D) and L929 cells (E) (n = 3). F, G) Calcein/PI staining was used to measure the viability of HUVECs and L929 cells (n = 3). Data were presented as mean ± SD. Statistical significance was determined using Student’s t test for A,B, one-way ANOVA followed by Tukey’s post hoc test for C-E. *p < 0.05, **p < 0.01, ***p < 0.001.


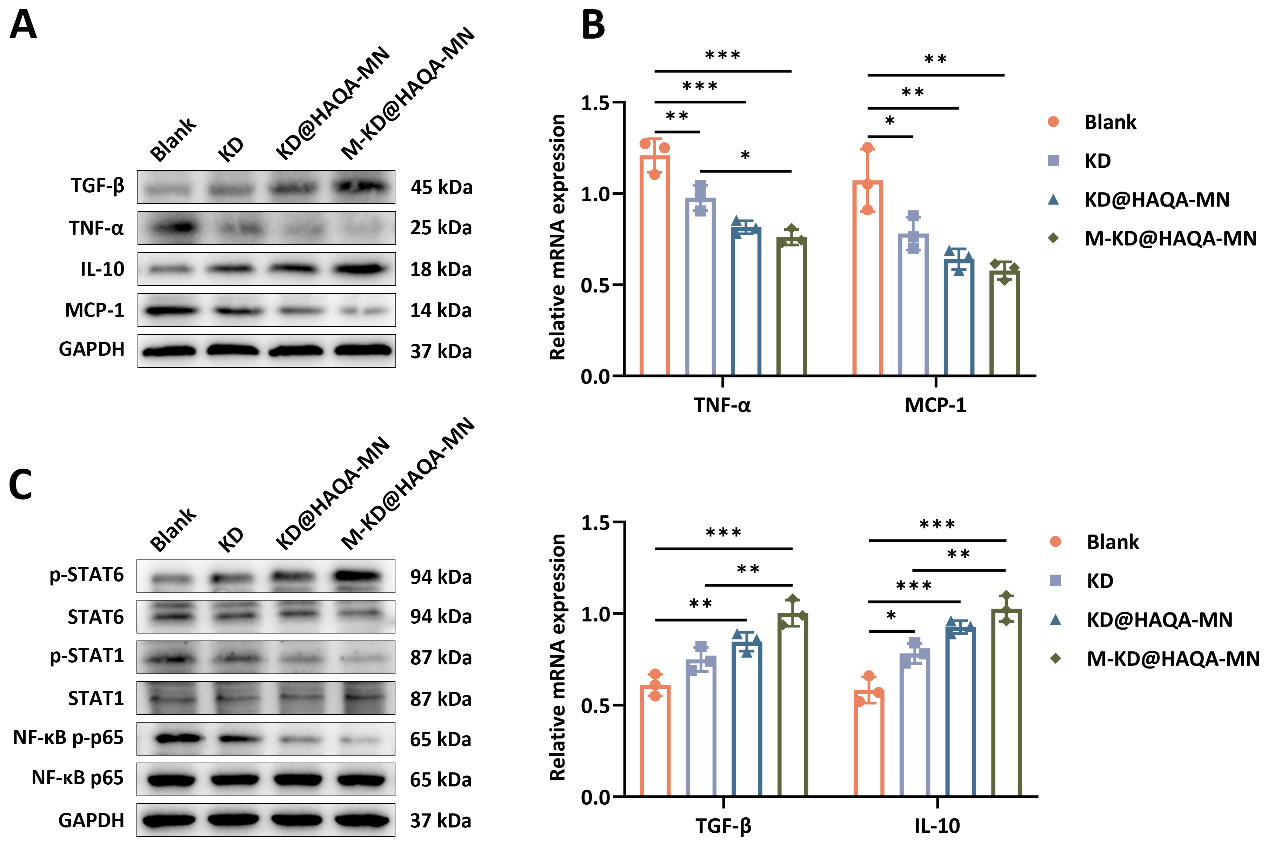


**Figure S15.** Inflammation profiles in the wound tissues following indicated treatments. A) Protein contents and B) mRNA levels of TGF-β, TNF-α, IL-10 and MCP-1 in the wound tissues at day 14 post-operation (n = 3). C) Western blot was performed to assess the activity of signaling molecule NF-κB, STAT6 and STAT1 in the wound tissues (n = 3). Data were presented as mean ± SD. Statistical analysis was performed using one-way ANOVA followed by Tukey’s post hoc test. *p < 0.05, **p < 0.01, ***p < 0.001.


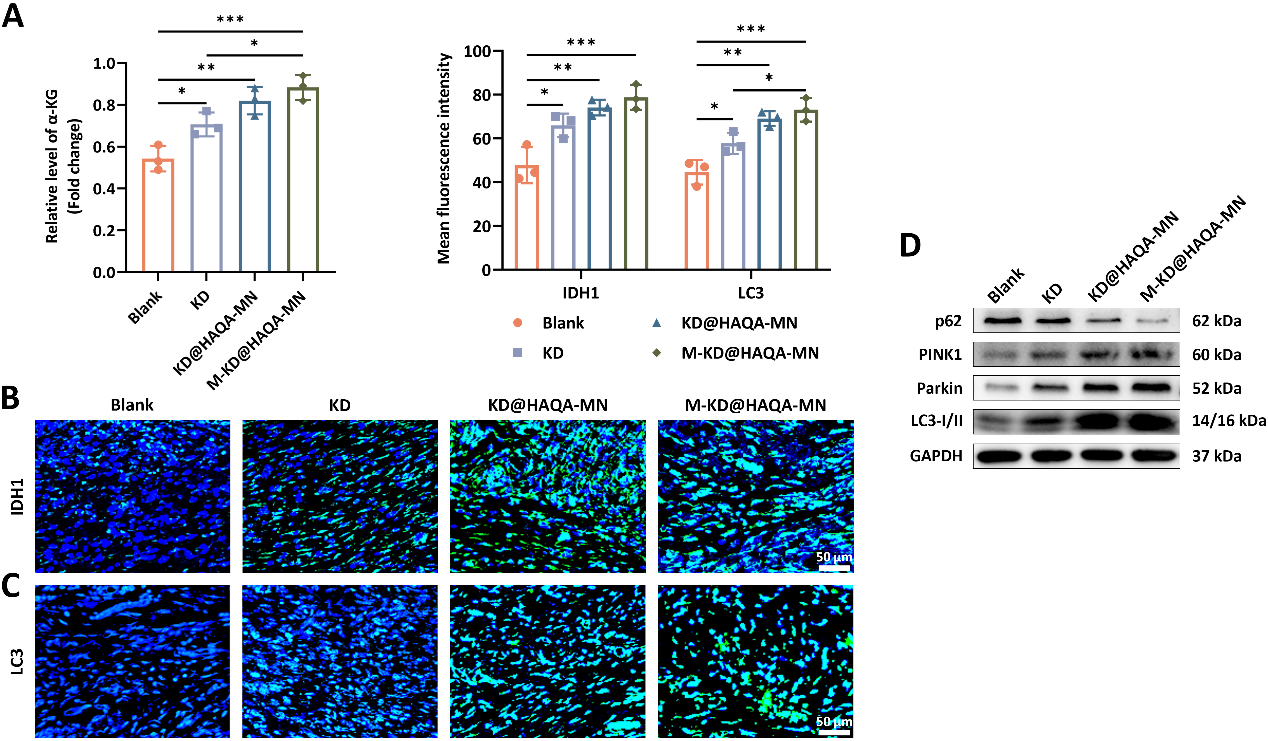


**Figure S16.** M-KD@HAQA-MN treatment affected TCA cycle and mitophagy in diabetic wound tissues. A) The relative level of α-KG in the wound area with different treatments (n = 3). B) IDH1 expression and C) LC3 level in the wound tissues was detected by immunofluorescence staining (n = 3). D) Proteins involved in Parkin-mediated mitophagy pathway was measured using western blot (n = 3). Data were presented as mean ± SD. Statistical analysis was performed using one-way ANOVA followed by Tukey’s post hoc test. *p < 0.05, **p < 0.01, ***p < 0.001.


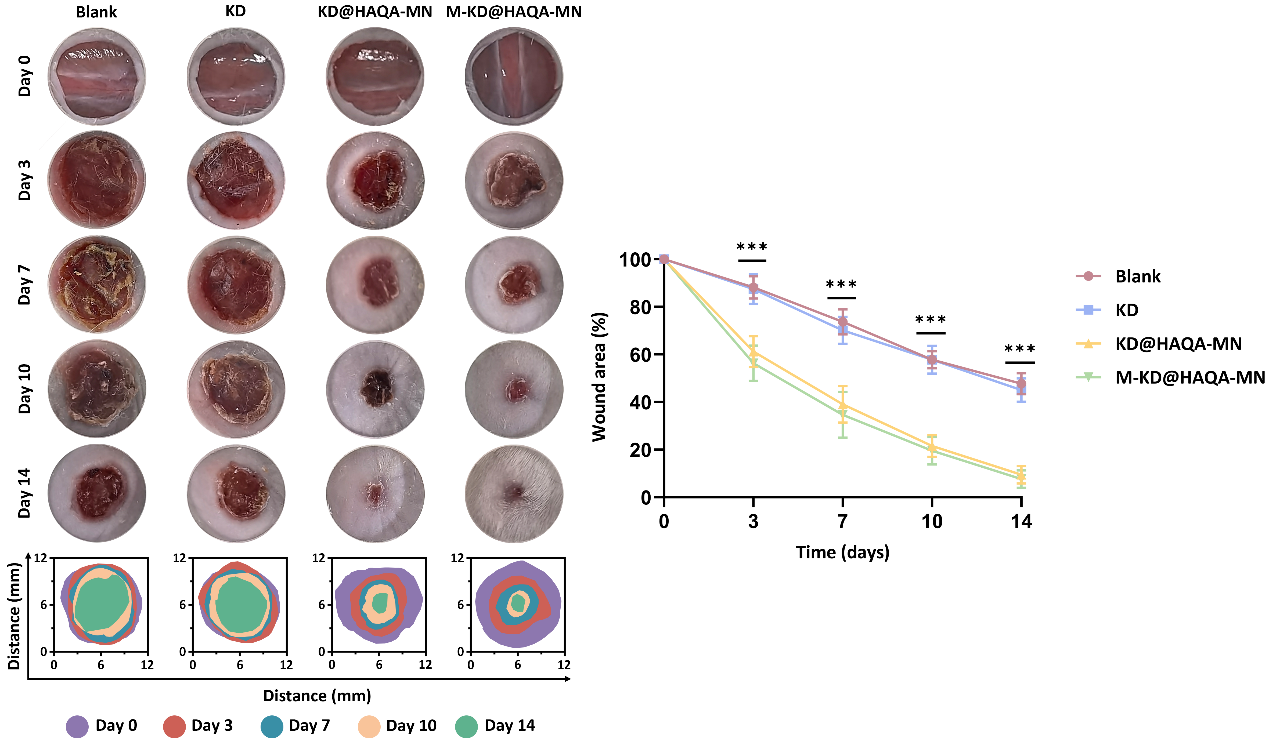


Figure S17. Treatment of the M-KD@HAQA-MN delivery system promoted infected wound healing of diabetic mice (n = 4). Data were presented as mean ± SD. Statistical analysis was performed using one-way ANOVA followed by Tukey’s post hoc test. ***p < 0.001.


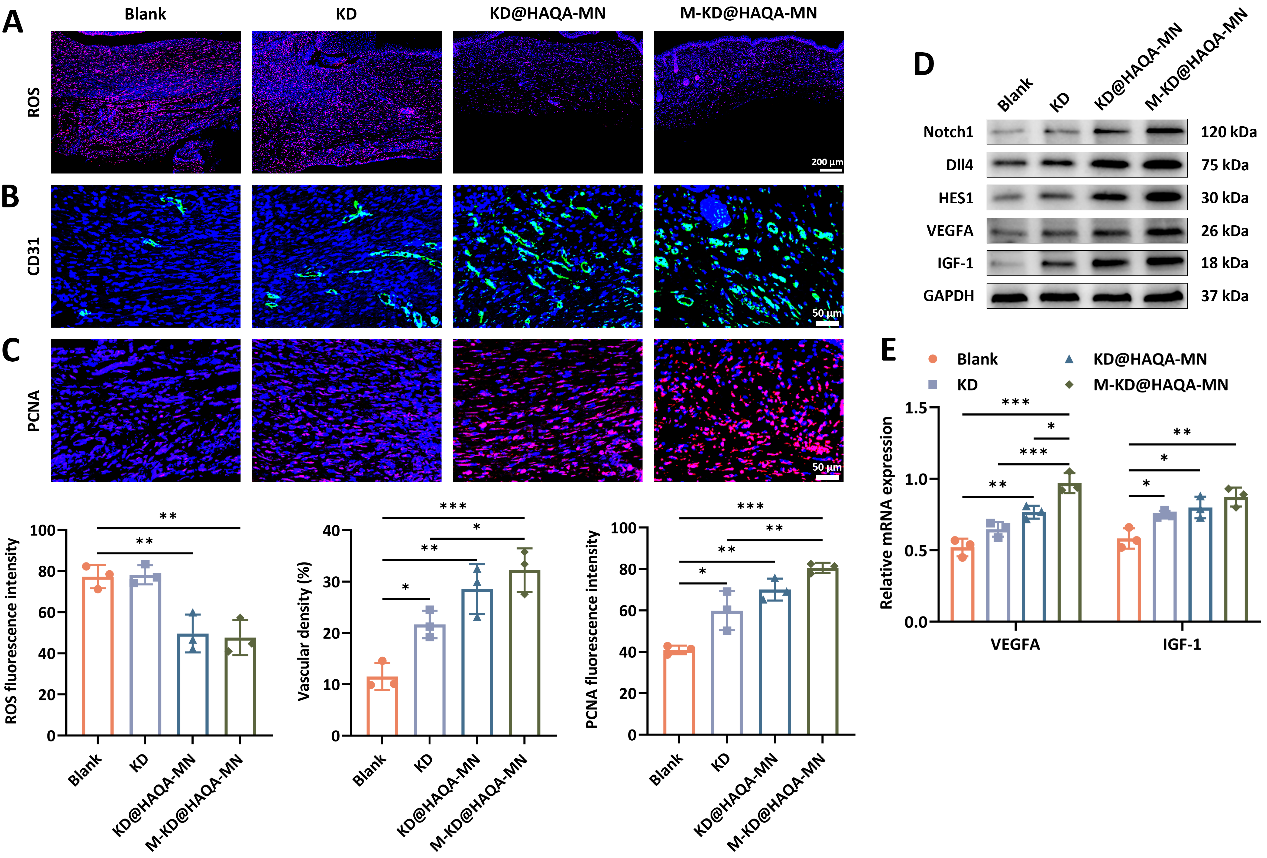


**Figure S18.** M-KD@HAQA-MN administration eliminated ROS and promoted angiogenesis in the diabetic wound tissues. A) The ROS level in the wound area was detected by DHE staining (n = 3). B) CD31 positive area and C) PCNA expression in the wounds tissues was visualized using immunofluorescence staining (n = 3). D) proliferation-related factor Notch1, Dll4, HES1, VEGFA and IGF-1 was detected by western blot (n = 3). E) qRT-PCR was employed to determine the level of VEGFA and IGF-1 mRNA (n = 3). Data were presented as mean ± SD. Statistical analysis was performed using one-way ANOVA followed by Tukey’s post hoc test. *p < 0.05, **p < 0.01, ***p < 0.001.


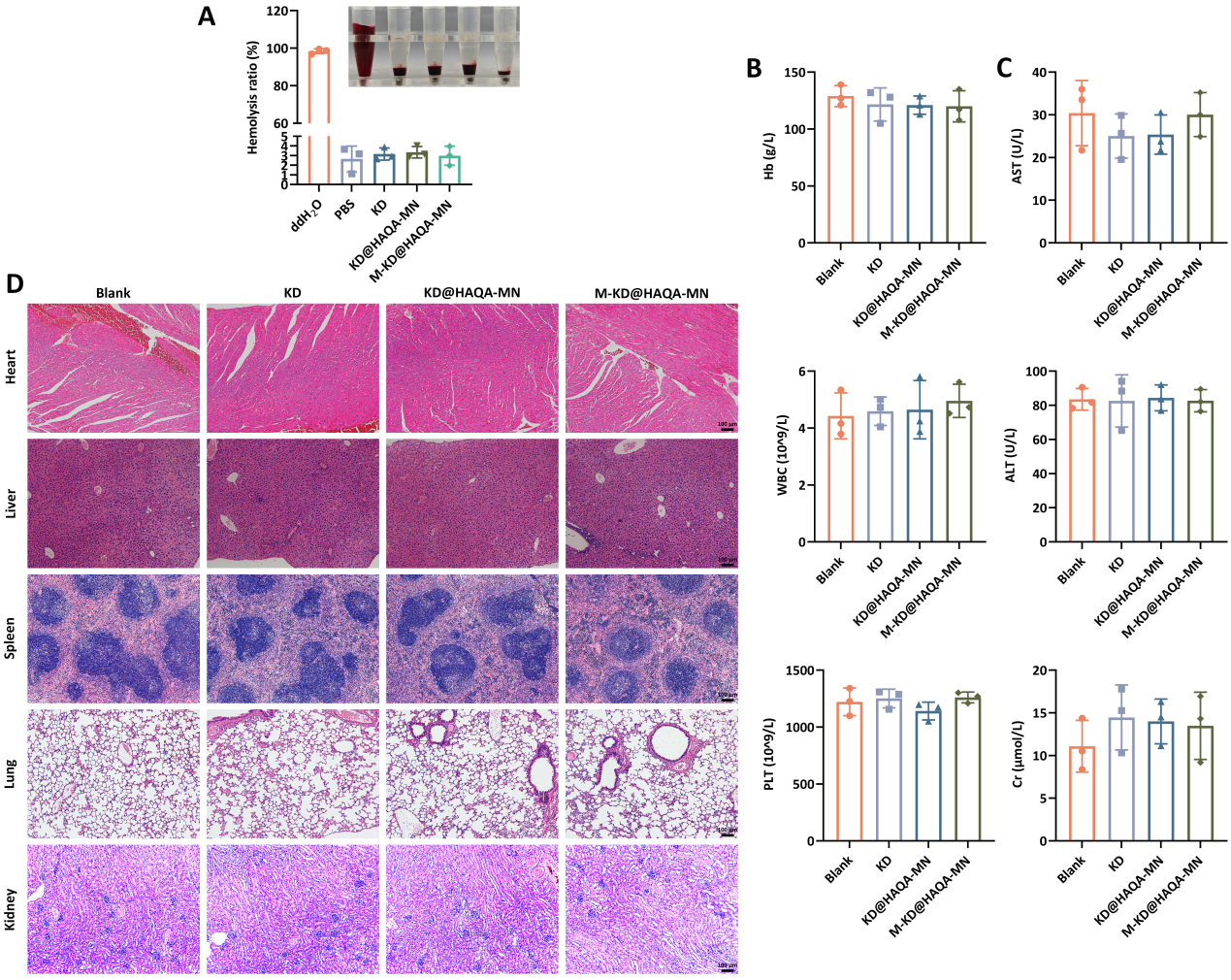


**Figure S19.** The biocompatibility of M-KD@HAQA-MN in vivo. A) Results of the hemolytic test (n = 3). B) Values of Hb, WBC and PLT and C) contents of AST, ALT and Cr in the Blank, KD, KD@HAQA-MN and M-KD@HAQA-MN groups (n = 3). D) The histological morphology of the heart, liver, spleen, lung and kidney with H&E staining (n = 3). Data were presented as mean ± SD. Statistical analysis was performed using one-way ANOVA followed by Tukey’s post hoc test.
